# Measuring rewilding progress

**DOI:** 10.1098/rstb.2017.0433

**Published:** 2018-10-22

**Authors:** Aurora Torres, Néstor Fernández, Sophus zu Ermgassen, Wouter Helmer, Eloy Revilla, Deli Saavedra, Andrea Perino, Anne Mimet, José M. Rey-Benayas, Nuria Selva, Frans Schepers, Jens-Christian Svenning, Henrique M. Pereira

**Affiliations:** 1 German Centre for Integrative Biodiversity Research (iDiv) Halle-Jena-Leipzig, Leipzig 04103, Germany; 2 Institute of Biology, Martin Luther University Halle-Wittenberg, Halle (Saale) 06108, Germany; 3 Durrell Institute of Conservation and Ecology, School of Anthropology and Conservation, University of Kent, Kent CT2 7NZ, UK; 4 Rewilding Europe, Toernooiveld 1, 6525 ED Nijmegen, The Netherlands; 5 Department of Conservation Biology, Estación Biológica de Doñana CSIC, Seville 41092, Spain; 6 Department Computational Landscape Ecology, UFZ – Helmholtz Centre for Environmental Research, Leipzig 04318, Germany; 7 Department of Life Sciences, University of Alcalá, 28805 Alcalá de Henares, Spain; 8 Institute of Nature Conservation Polish Academy of Sciences, Av. Mickiewicza 33, 31-120 Krakow, Poland; 9 Department of Bioscience, Center for Biodiversity Dynamics in a Changing World (BIOCHANGE), Aarhus University, Ny Munkegade 114, 8000 Aarhus C, Denmark; 10 Section for Ecoinformatics and Biodiversity, Department of Bioscience, Aarhus University, Ny Munkegade 114, 8000 Aarhus C, Denmark; 11 Centro de Investigação em Biodiversidade e Recursos Genéticos (CIBIO), Universidade do Porto, 4485-661, Vairāo, Portugal

**Keywords:** biodiversity, ecological processes, ecosystem integrity, ecosystem management, monitoring, restoration

## Abstract

Rewilding is emerging as a promising restoration strategy to enhance the conservation status of biodiversity and promote self-regulating ecosystems while re-engaging people with nature. Overcoming the challenges in monitoring and reporting rewilding projects would improve its practical implementation and maximize its conservation and restoration outcomes. Here, we present a novel approach for measuring and monitoring progress in rewilding that focuses on the ecological attributes of rewilding. We devised a bi-dimensional framework for assessing the recovery of processes and their natural dynamics through (i) decreasing human forcing on ecological processes and (ii) increasing ecological integrity of ecosystems. The rewilding assessment framework incorporates the reduction of material inputs and outputs associated with human management, as well as the restoration of natural stochasticity and disturbance regimes, landscape connectivity and trophic complexity. Furthermore, we provide a list of potential activities for increasing the ecological integrity after reviewing the evidence for the effectiveness of common restoration actions. For illustration purposes, we apply the framework to three flagship restoration projects in the Netherlands, Switzerland and Argentina. This approach has the potential to broaden the scope of rewilding projects, facilitate sound decision-making and connect the science and practice of rewilding.

This article is part of the theme issue ‘Trophic rewilding: consequences for ecosystems under global change’.

## 1. Introduction

Increasing global consumption of natural resources, population growth and rapid environmental changes have led to widespread loss and degradation of ecosystems [1–3], with potentially serious consequences for biodiversity and human well-being. These global changes involve different degrees of simplification and homogenization of natural systems, from defaunation that cascades through trophic networks reducing ecosystem function [4] to extreme depletions of biodiversity in intensively transformed ecosystems as land-use changes proceed [5].

Rewilding is emerging as a promising restoration strategy in a human-dominated world to promote self-sustaining ecosystems and enhance the conservation status of biodiversity [6–9]. This concept is gaining momentum and becoming increasingly influential in restoration ecology and conservation science. Rewilding initiatives are leading to the emergence of an empowering environmental narrative, which has been coined ‘Recoverable Earth’ [10], placing the restoration of ecological systems at the centre of societal change. Rewilding is viewed as a possible pathway societies can take towards sustainability [11], because it has the potential to generate co-benefits that extend beyond natural heritage conservation (e.g. [12–14]).

Recent studies describe rewilding as a nature restoration action that emphasizes the dynamic character of ecosystems and that explicitly acknowledges the role of reducing human forcing of the system [14,15], i.e. human control or influence on the system. Furthermore, rewilding initiatives aim to give a response to public demand for a sense of ‘wildness’ [16], strongly supporting the emotional value of exposure to perceived untamed nature. With the number of rewilding initiatives growing [10,17,18], it is imperative that monitoring and assessment plans are developed and adopted. Overcoming the challenges in monitoring and reporting on rewilding projects would improve the practical implication of rewilding and maximize its conservation and restoration outcomes. In this study, we focus on the ecological attributes of rewilding, whereas there is a parallel project needed to unpack socio-economic ones. We adopt the definition of rewilding as the process of restoring the structural and functional complexity of degraded ecosystems while gradually reducing the human influence [15]. Underpinned by this idea, we aim to provide a framework for measuring and monitoring the ecological integrity of ecosystems and reducing the human forcing on these (thereafter referred to as ‘measuring rewilding progress’).

Approaches to monitor restoration progress and success rely on the quantification of indices of recovery progress [19,20], recovery completeness [21] or both [22], which compare degraded, restored and intact reference ecosystems. In all these cases, a key step in assessing restoration progress is finding and agreeing on a reference ecosystem, though increasingly considering environmental change. Furthermore, organizations such as IUCN and the Society for Ecological Restoration (SER) provide guidelines to audit restoration projects [23,24]. One of the key principles underpinning these guidance documents is restoring the ecological integrity of ecosystems. To that end, in the IUCN guidance, ecological integrity is mainly assessed by monitoring the structure, function and composition of an ecosystem (https://www.iucn.org/content/ecological-restoration-protected-areas-principles-guidelines-and-best-practices) [23], whereas the SER guidelines propose monitoring the absence of threats, physical conditions, species composition, structural diversity, ecosystem functionality and external exchanges (https://www.ser.org/page/SERStandards) [24]. However, there is no restoration monitoring framework at present that combines the human forcing on natural processes and the changes in the ecological integrity of ecosystems.

Within this restoration context, rewilding is aligned with newer visions of restoration (e.g. ‘Restoration v. 2.0’ [25] or ‘open-ended restoration’ [26,27]) that are process-oriented and recognize the dynamism of landscapes and of ecological processes [25,28,29]. These approaches use historical knowledge as a guide and not as a template for determining restoration goals, highlight the continuing dynamic nature of the ecosystem as an embedded restoration goal, accept multiple potential trajectories for ecosystems, emphasize process over structure and composition, embrace pragmatic approaches to address human livelihoods and cultural needs and are particularly useful from landscape to larger scales [25–27,30,31]. Our framework for measuring rewilding progress does not conceptually depart from these guiding principles but it rather emphasizes some specific aspects mentioned above and further developed in the next section.

Here, we present a novel approach on how to measure and monitor rewilding progress. We devised a bi-dimensional framework to assess the recovery of processes and their natural dynamics through (i) decreasing direct human inputs and outputs of materials into the system and (ii) restoring the ecological integrity of ecosystems [15,32]. This framework also allows the comparison of rewilding progress between areas. For this, we propose the use of pressure and state variables and associated indicators describing both the human control over the system and the ecosystem's ecological integrity to measure its position along a naturalness gradient. This approach has the potential to broaden the scope of ecological restoration, facilitate sound decision-making and connect the science and practice of rewilding.

## 2. A rewilding assessment framework

### (a) Conceptual framework

**Figure 1.**
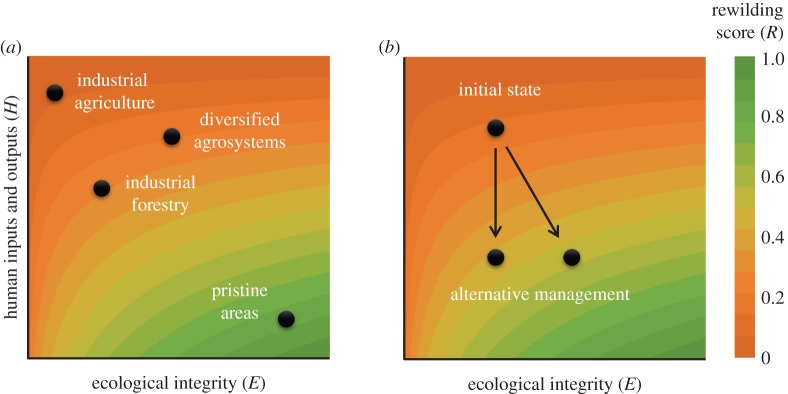
Bi-dimensional space representing the condition of the system along axes of human input and output forcing (*H*) and ecological integrity of ecosystems (*E*). Background colours represent the values of the rewilding score quantified through equation (2.3). (*a*) Conceptual illustration showing the position of common land uses in this bi-dimensional naturalness space. (*b*) Scheme of how changes in either dimension can lead to changes in overall system condition, although improvements in both dimensions are typically required to maximize the rewilding score. (Online version in colour.)

We assume that the condition of ecosystems is a function of the intensity of human forcing over natural processes and of the system's ecological integrity [15]. We defined a bi-dimensional space to capture these two dimensions (figure 1) and identified a set of pressure and state variables contributing to each of the two axes (table 1). The position of the system in that space can change as a result of restoration actions, thus allowing the measurement and monitoring of rewilding through time.

**Table 1.** List of pressure and state variables and indicators proposed for measuring rewilding progress and associated restoration actions. The scores are assigned in a continuous scale from 0 to 1. Reference values provide guidance for expert assessments (further details in electronic supplementary material, table S1). Effectiveness of restoration actions (EF) for achieving rewilding objectives—namely, to restore trophic processes, landscape connectivity, natural disturbance regimes and/or biodiversity—was based upon the review of evidence (electronic supplementary material, table S2) inspired by the Conservation Evidence approach (www.conservationevidence.com) [33], where EF = 0: no evidence or unknown effectiveness of restoration action; EF = 1: likely to be ineffective; EF = 2: trade-off between benefit and harm; EF = 3: likely to be beneficial; EF = 4: beneficial.

pressure and state variables	indicator	score	restoration action	EF
**direct human inputs and outputs**				
artificial feeding of wildlife	is artificial feeding of animals allowed, and how influential is it on ecological processes?	0—no artificial feeding; 0.5—some type of artificial feeding is provided at levels unlikely to significantly affect animal movements, species diet, seed dispersal and other ecological processes; 1—high levels of artificial feeding and/or evidence for feeding affecting ecological processes (e.g. artificial food is an important component in the diet of a species)	reduce to a minimum or eliminate any type of artificial feeding that may potentially influence animal behaviour and ecology	2
population reinforcement	have any animals been (re-)introduced into the area within the last years?	0—no population reinforcement at least during the last year; 0.5—species populations of conservation concern sporadically reinforced to improve their conservation status; 1—regular to intensive population reinforcement for the conservation of populations that would otherwise decline, or reinforcement of non-declining populations or populations of no conservation concern	establish self-sustaining populations so that further population reinforcement is unnecessary	0
agricultural production	cropland area and farming intensity	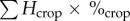 where: %_crop_ = proportion of the total rewilding area devoted to cropland, *H*_crop_ = 0—no harvested or fallow for at least 5 years (i.e. land abandonment); 0.5—cropped and harvested under traditional, extensive farming practices; 1—intensive harvesting, every year	reducing farming intensity and extent (land abandonment)	2
forestry production	forest area dedicated to forestry production (e.g. wood, timber, pulp) and forest management intensity	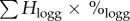 where: %_logg_ = proportion of the total rewilding area devoted to production forestry, *H*_logg_ = 0—no logging for at least 5 years; 0.5—Selective logging; 1—clear-cut logging	cessation and/or reduced harvesting. This should prioritize old-growth forest	4
grasslands production	grassland area dedicated to hay and livestock production and intensity of production. Free-roaming wild ungulates do not count towards this indicator	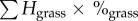 where: %_grass_ = proportion of total rewilding area devoted to managed grasslands, *H*_grass_ = 0—no harvesting for at least 5 years (land abandonment); 0.5—mowed under traditional, extensive farming practices; 1—intensive harvesting or very high livestock stocking densities	reducing mowing and ploughing in grasslands, and reducing livestock intensity	2
mining	area devoted to mining and intensity of the impacts of mining on the ecosystem	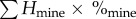 where: %_mine_ = proportion of total rewilding area devoted to open mining, *H*_mine_ = 0—No mining for at least 5 years; 0.5—mining with non-destructive production practices (e.g. artisanal mining) and strict regulation and mitigation of pollution; 1—intensive mining with destructive mining practices and clear evidence of degradation	reduce mining and mining impacts	3
harvesting of terrestrial wildlife	is hunting allowed? To what extent is the ecosystem affected by hunting?	0—no hunting; 0.5—low levels of hunting unlikely to significantly affect the growth rates of wildlife populations, animal movements, or other species with which hunted species interact; 1—high levels of hunting and/or probable or demonstrated effects on the growth rates and/or the population structure of harvested populations or species interactions	restriction of hunting	4
harvesting of aquatic wildlife	is extractive fishing allowed? To what extent is the ecosystem affected by extractive fishing?	0—no extractive fishing; 0.5—fishing only in artificial ponds or low levels of extractive fishing unlikely to significantly affect the growth rates of wildlife populations, animal movements, or species with which fished species interact; 1—high levels of extractive fishing and/or probable or demonstrated effects on the growth rates and/or the population structure of harvested populations or species interactions	restriction of extractive fishing	4
carrion removal	does regulation permit leaving medium and large carcasses in the field?	0—carcasses from wild animals and extensive livestock are left in the field; 0.5—carcasses of wildlife are left in the field, those from extensive livestock are removed; 1—all carcasses are removed from the field	legislative change to permit leaving carcasses in the field	2
deadwood removal	is deadwood (dead trees and woody debris) removed?	0—no deadwood removal; 0.5—Low levels of deadwood removal (e.g. on roads and footpaths) unlikely to affect disturbance regime, animal movements and other ecological processes significantly; 1—high levels of deadwood removal	allowing deadwood to remain in the forest	3
**ecological integrity**				
disturbance regimes				
natural avalanche and/or rock slide regimes	are avalanche and/or rock slide regimes regulated?	0—regulation of avalanches and/or rock slides across the whole rewilding area; 0.5—avalanches and/or rock slides only in certain places with risk for human life; 1—no regulation of the avalanche and/or rock slide regime	restoring the natural regime of avalanches and rock slides	3
natural fire regimes	are there deviations of the natural fire regime due to human pressures (this might be in either direction, i.e. fire suppression, or prescribed burning)?	0—fire regime heavily modified by human intervention including both artificial burning and/or fire suppression; 0.5—artificial burning and/or fire suppression is very localized and only cause minor ecological impacts; 1—there are no deviations of the natural fire regime	restoring the natural regime of fires, including restoration and/or natural regeneration of native fire-dependent vegetation	3
natural hydrological regimes	are hydrological regimes (including flood regimes) heavily modified?	0—high regulation of the natural hydrological regime; 0.5—dams in place, but cause only minor impacts on the overall hydrological regime; 1—no regulation of the hydrological regime	restoring the natural hydrological regime (e.g. removing dykes, channels, dams)	3
natural pest regimes and mortality events	are natural pest regimes and mortality events regulated? Are management actions implemented after mortality events (e.g. storms, pests)?	0—management actions implemented to avoid pests (e.g. pesticide or vaccination use) or after mortality events (e.g. salvage logging, removal of burnt wood); 0.5—low levels of management to avoid pests or after mortality events, unlikely to affect disturbance regime, animal movements and other ecological processes significantly; 1—no management to avoid pests or after mortality events	passive restoration after mortality events (e.g. avoiding pesticide use) and avoid acting against natural pests	2
landscape connectivity and composition				
terrestrial landscape fragmentation	to what extent is the landscape fragmented by human infrastructure? What is the effective mesh size [42] of the rewilding area?	0—landscape highly fragmented (fully covered with heavily used infrastructure); 0.5—landscape crossed by low-traffic roads and infrastructure; 1—landscape not fragmented	restoring connectivity including removing, bundling or reducing the extent of linear transport infrastructure and built-up areas (excluding abandoned buildings)	4
aquatic landscapes fragmentation	to what extent are migratory processes in river systems allowed?	0—fish migration fully impeded; 0.5—dams in place but alternative migration routes or fish cannons provided; 1—no regulation of fish migration	restoring aquatic habitat connectivity	4
spontaneous vegetation dynamics	what is the state of natural regeneration?	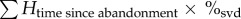 where: %_svd_ = proportion of area where spontaneous vegetation dynamics are allowed, *H*_time since abandonment_ = 0.1—early successional stages (e.g. less than 50 years); 0.5—(50–200 years); 1—last successional stages adapted to each ecological region or biome (e.g. greater than 200 years old-growth forest). For time since abandonment, allocate discrete scores (i.e. not on continuous scale)	allowing natural succession	4
harmful invasive species	what is the impact of harmful invasive species on the rewilding area?	0—very severe impacts of invasive species on ecological communities in the rewilding area; 0.5—impacts of invasive species within small, localized communities within the rewilding area; 1—no major invasive species present	removal of harmful invasive species	3
trophic processes				
terrestrial large-bodied fauna (greater than 5 kg)	species composition of large-bodied (greater than 5 kg) species	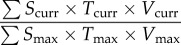 where: *S* is the space occupied by the species in the area, estimated from 0–1; *T* is the percentage of the time in a year that species are present in the area they occupy (estimated 0–1, except for migratory species that if present should score 1); *V* is the viability of the population to which the individuals of the species belong that can be larger than the focal area (estimated 0–1); *curr* denotes the values for each species at a given time; and *max* denotes the maximum possible value for each variable for that species (always equals 1)	recovery of large-bodied species	3

In this framework, both axes capture changes in the natural condition of the system at different temporal scales. The axis of human inputs and outputs (*H*) captures the pressures of direct human forcing on the ecosystem at the time of measurement; thus, changes in management regimes will immediately be captured by changes in the rewilding score on this axis. This metric of human control can be considered an application of the ‘cultural energy’ framework in [34], whereby the ‘unnaturalness’ of a system can be quantified by the degree of human-associated energy inputs required to maintain the ecological system in its current state; however, instead of measuring the actual energy inputs, we propose measuring indicators of human inputs and outputs that can be readily assessed by practitioners without specialized knowledge or data. On the other hand, the ecological integrity axis (*E*) is affected by human legacy effects on ecological composition, structure and functions. Hence, there will be temporal lags—from days to even centuries—between the implementation of restoration actions and the resulting increase in the integrity of the system [21,35]. In other words, these human legacies (e.g. caused by roads or dams) and the natural dynamics of ecosystems including species colonization and extinction rates constitute the ecological inheritance of the ecosystem and will determine its trajectory into the future [26]. Uncovering these human legacies contributes to explaining the distinctive characteristics of a rewilding area, identifying constraints or challenges in shaping the ecosystem in the future and planning active restoration actions (e.g. road or dam removal).

The human forcing on natural processes and ecosystem dynamics is defined here as a function of the direct human inputs and outputs of material into the systems that are linked to today's management:
2.1
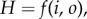
where *i* corresponds to material inputs into the system (e.g. baiting of wildlife) and *o* to material outputs (e.g. timber production, hunting, mining). While some indicators combine both inputs and outputs (e.g. agricultural production), we do not quantify inputs and outputs separately. Importantly, this axis also captures impacts from management activities (e.g. removing deadwood for pest control or wildlife population control) and, in some cases, conservation management activities with a direct influence on the system dynamics, such as population reinforcements that are expected to have a limited duration. That said, certain rewilding projects might require an initial level of active restoration to overcome constraints that prevent full restoration of natural processes that eventually will translate into an increase of ecological integrity.

While most approaches to monitoring restoration progress focus on the composition, structure and function of ecosystem [24,36], we consider that the ecological integrity of ecosystems is defined according to three core principles critical for self-sustaining ecosystems (Perino *et al*., under review): namely to (i) allow for natural stochasticity and disturbances influencing ecological processes [37], (ii) increase landscape connectivity of terrestrial and aquatic systems [38] and (iii) enhance completeness of degraded trophic networks [8]. For instance, it has been shown that natural disturbances contribute to ecosystem-level processes (e.g. primary production, sedimentation, ecological succession), species interactions (e.g. trophic relationships), structural effects (e.g. development of mosaics of habitats) and allowing intraspecific processes (e.g. migration in rivers) [37]. Animal movements, and therefore connectivity, are essential for ecosystem functioning because they act as mobile links and mediate key processes such as seed dispersal, food web dynamics and metapopulation and disease dynamics, which have been shown to provide sources for reorganization after major disturbance events [39,40]. Likewise, recovering diverse species communities requires maintaining viable populations and enabling the recovery of declining and depleted populations, which are typically at higher trophic levels.

We adopt these three guiding principles as normative standards for measuring the ecological integrity of the system in the *E* axis:
2.2
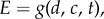
where *d* represents the naturalness of disturbances and stochastic events, *c* the connectivity of terrestrial and aquatic systems and *t* the composition and complexity of the trophic network. The value of these three components should be increased in a rewilding process. By considering the interaction among these ecosystem components, this approach allows us to gauge the ability of an ecosystem to support and maintain ecological processes and biodiversity as well as to adapt to ongoing and future changes [41]. Human legacy effects on ecosystem dynamics, for example harmful invasive species competing with ecologically important native species or altering ecological processes, are accounted for in this axis.

Within this framework, people can exist and thrive in the rewilding system as long as their activities do not compromise the progress towards decreasing the human forcing of ecological processes and increasing the ecological complexity of the system. In other words, there is space for human activities such as non-extractive industries and managed eco-tourism.

### (b) Operationalizing the framework

The rewilding assessment framework was developed combining expert knowledge, analysis of data and feedback from stakeholders including conservation and rewilding practitioners, in an attempt to balance between the reliable recording of ecological changes (i.e. how accurately the score reflects the natural condition of the system) while ensuring real-world applicability (i.e. the degree to which the approach could be routinely used with the best available knowledge or data). To select experts for each case study, we first identified the type of expertise required to monitor rewilding progress including an understanding of the complexity of different ecosystem components and familiarity with spatial information. Then, we selected experts with demonstrated background on the study system in the field (according to the extent and duration of the rewilding project), and the professional connection to conservation or restoration agencies and organizations [43].

Our set of pressure and state variables and indicators allows measuring the rewilding progress on a particular site, namely the rewilding project. This focal unit may be defined at any spatial and temporal extent. Nevertheless, we recognize that human infrastructure and activities beyond the spatial boundaries of the rewilding area might interfere with the recovery of its naturalness, in particular through their impact on connectivity and dispersal. In addition, because of the slow speed of expected ecosystem recovery and the long-term nature of rewilding projects, 5-year or longer monitoring cycles are recommended [27].

To select variables and indicators, we drew up a list of the major human inputs and outputs into ecosystems, and of potential indicators that could be used to describe the naturalness of disturbance regimes, landscape connectivity and composition, and trophic processes. We then revised the indicators to ensure that they were conceptually independent and that they were implementable by practitioners without specialist knowledge (e.g. the deviation of the existing vegetation community from the pre-human baseline vegetation community was dropped because assessing this baseline with any degree of certainty would require intensive paleo-ecological analysis). In addition, following best practices, indicators should ideally be (i) feasible to monitor; (ii) useful at multiple spatial and temporal scales; (iii) practical to implement, without prohibitive technical or financial requirements; (iv) respond predictably to human impact; and (v) represent a causal impact on the desired outcome [44–46]. It was also essential that practitioners can quantify these indicators in a standardized and replicable manner across a range of scenarios and contexts.

We assembled a suite of 18 indicators tied to particular restoration actions, from passive or non-intervention to active management (table 1; electronic supplementary material, table S1). These include a combination of quantitative and qualitative indicators, with the emphasis given to indicators that best navigate the trade-off between simplicity and accuracy. We adopted quantitative indicators where we felt the technical capabilities required were realistic for practitioners. For the qualitative indicators, we adopted an approach that used a combination of multiple qualitative indicators to reduce biases affecting any individual indicator.

In the bi-dimensional rewilding space (figure 1), it is possible to compare systems described under the same set of components and to monitor changes in time. The framework informs on the system's condition at a certain time relative to the maximum plausible long-term improvement that could be achieved for each variable in terms of maximizing ecological integrity. To do so, we give a score (*S*) to each variable. The scores are described on a continuous 0–1 scale, in which 1 represents the maximum intensity of human forcing (*H*_max_; for variables in the *H* axis) or the maximum ecological integrity (*E*_max_; for state variables in the *E* axis, e.g. hydrological regime is not regulated), and 0 represents an area without human inputs or outputs into the system (*H*_min_) or with minimum influence of human legacy effects on ecosystem composition, structure and functions (*E*_min_), respectively. Reference values for each indicator are proposed in table 1 and electronic supplementary material, table S1, which also provide guidance for expert assessments.

The score for each of the components of the framework is calculated as the arithmetic average standardized scores of the variables within such component. Thus, a normalized score on a continuous 0–1 scale is obtained for the human inputs and outputs into the system (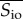), the naturalness of disturbance regimes (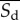), the landscape connectivity (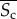) and the trophic complexity (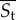). Next, the position of a given system in the *N* axis is calculated as the geometric mean of the scores for the naturalness of disturbance regimes, the landscape connectivity and the trophic complexity. This integration based on the geometric mean emphasizes the critical role of the interactions among the three ecosystem components in rewilding.

Finally, the values for the *H* and *E* axes describe the position of a given system at a snapshot in time. The combination of both values yields a total cumulative rewilding score (*R*):
2.3
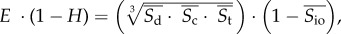
where *R* values range from 0 to 1. For a particular site, a higher positive change in *R* means a higher success of rewilding, i.e. a reduction in human forcing over natural processes and/or increase in ecological integrity. Given that they are based on a standardized set of indicators, *H*, *E* and *R* can be compared across diverse rewilding projects. However, a complementary set of additional indicators could be tailored specifically for any given rewilding project to capture the local nuances. In this case, however, the general and components scores may no longer be comparable between systems.

### (c) Evidence-based restoration actions for rewilding projects

Rewilding initiatives need to move beyond anecdote, personal experience, expert criteria and conventional wisdom, towards a more systematic appraisal of evidence collected by practitioners tackling a given restoration action. Here, together with the list of pressure and state variables and indicators, we provide a list of management activities for rewilding based upon the review of evidence inspired by the Conservation Evidence approach (www.conservationevidence.com) [33]. Thus, for each variable in the framework, we identified the key restoration action that could be implemented in order to increase the score for that variable.

We gathered evidence on the effectiveness of 16 restoration actions by reviewing 137 primary studies from key scientific journals for each action. We used Web of Science and Google Scholar to identify and review primary studies evaluating the evidence for each action where available. When no reviews were identified, we searched for studies on each topic published since 2014 and used these publications to identify further relevant studies for evaluating each action. Next, we reviewed the collated evidence and added further key studies when required. We summarize the evidence for each restoration action in electronic supplementary material, table S2, and scored these activities from 0 to 4 according to the effectiveness of the intervention (e.g. 2—‘trade-off between benefit and harm’, 4—‘beneficial’). We conceive these evidence syntheses as a key first step towards systematic revision of evidence for the effectiveness of each restoration action in the context of rewilding projects.

### (d) Case studies

**Figure 2.**
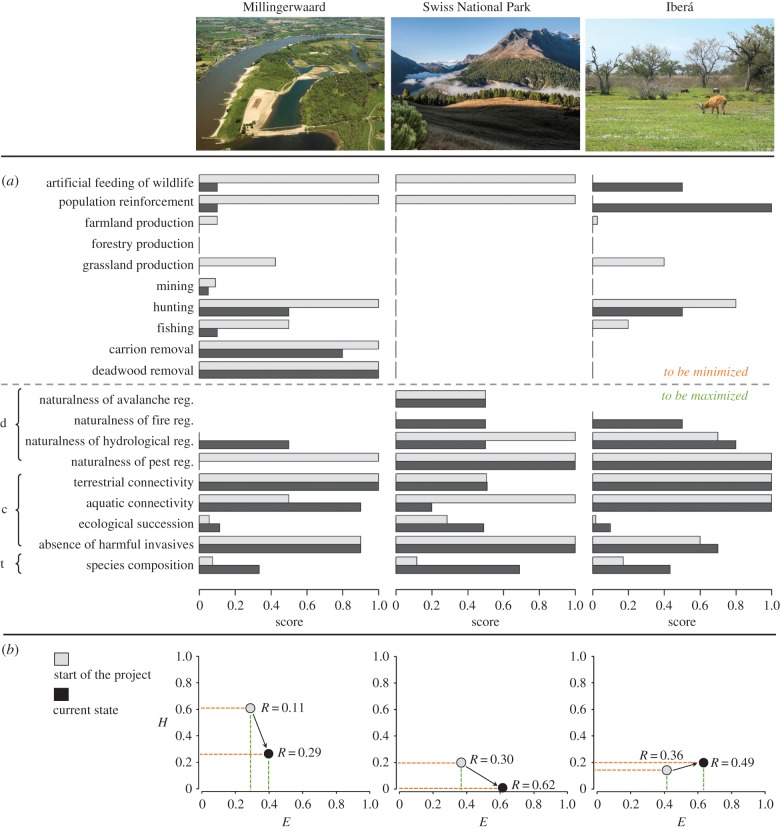
Panel showing the results of applying the rewilding assessment framework to three projects, namely the Millingerwaard project (the Netherlands); the Swiss National Park (Switzerland); and the Iberá project (Argentina). (*a*) Scores obtained for the variables at the beginning of the project and at present. A description of the variables and indicators is available in table 1 and electronic supplementary material, table S1. (*b*) Representation of the estimated scores of direct human inputs and outputs (*H*) and ecological integrity of ecosystems (*E*) in the bi-dimensional framework for each case study. *d* variables represent the naturalness of disturbances and stochastic events, *c* variables represent landscape composition and connectivity and *t* variable represents the trophic complexity. The arrows indicate the trajectory of change from the beginning of the projects to present. The rewilding score (*R*) is placed next to each point in time and has been calculated on the basis of the scores shown in (*a*). Photographs courtesy of Rijkswaterstaat, SNP/H. Lozza and N. Fernández. (Online version in colour.)

To illustrate and test the framework, we applied the assessment to three flagship restoration projects with very different characteristics: the rewilding area of Millingerwaard in a highly urbanized landscape 10 km outside Nijmegen (the Netherlands); the Iberá Project (Argentina), which is one of the largest naturalized inland wetland systems in South America [47]; and the Swiss National Park (southeast Switzerland), which has been managed to minimize the human control of ecological processes for over a century (table 2 and figure 2). As the knowledge required was highly context-specific, we contacted one practitioner per study area. They were invited to fill in a questionnaire that compiled the indicators previously mentioned. The expert provided a score for each indicator at the beginning of the rewilding project and at present. The encoding schemes are documented by a guidance document that includes an extended description of the indicators, reference values and examples (electronic supplementary material, table S1). This makes it possible to scrutinize the methods and to reproduce and validate the assessments. Finally, reception of the questionnaire was followed up by an interview to ensure a consistent assignment of scores.

**Table 2.** Overview of the three rewilding projects used as case studies sorted by increasing size.

project	ecological description	initial sources of degradation	main restoration actions developed	ecological responses
Millingerwaard (The Netherlands) Project start: 1990 Size: 700 ha	naturalized floodplain, matrix of grasslands, cropland and wetlands	use of land for agriculture. dykes to reduce the flooding risk	dyke removal, restoration of natural hydrological regime, release of Konik horses (*Equus ferus*) and Galloway cattle (*Bos taurus*) to promote natural grazing, reversion of agricultural land to unmanaged. Reintroduction of beaver (*Castor fiber*) and Atlantic sturgeon (*Acipenser oxyrinchus*)	recovery of riverine vegetation and ecological communities. Surplus of horses and bovines relocated away annually. Recovery of ecosystem engineers such as wild boar (*Sus scrofa*) and other native predator species including otter (*Lutra lutra*) and white-tailed eagle (*Haliaeetus albicilla*)
Swiss National Park (Switzerland) Project start: 1914 Size: 17 000 ha	large extent of alpine and subalpine habitats, including *ca* 30% coniferous forest and *ca* 20% grasslands. The area captures full successional gradient from short-grass pastures to Swiss stone pine stands (*Pinus cembra*)	before 1914, the area was widely used for timber production and alpine farming	IUCN category 1A nature reserve, affording strict protection to all natural ecological processes in the park (except fire, which is suppressed in most of the park). Reintroductions of the ibex (*Capra ibex*) in 1920s–1930s and the bearded vulture (*Gypaetus barbatus*) in 1990s–2000s	uninhibited succession across the park, but subalpine grasslands are kept open at least partly through natural browsing [48]. Viable populations of numerous large herbivores, including red (*Cervus elaphus*) and roe deer (*Capreolus capreolus*) and chamois (*Rupicapra rupicapra*), and of smaller predators such as the golden eagle (*Aquila chrysaetos*). Sporadic presence of lynx (*Lynx lynx*) and brown bear (*Ursus arctos*)
Iberá (Argentina) Project start: 1999 Size: 150 000 ha	Rain-fed wetland, with *ca* 60% permanently flooded. Matrix of grasslands and forests [49]	hunting of large terrestrial animals to extinction, grazing by livestock, burning of rangelands and logging of trees for timber	multiple reintroductions including giant anteaters (*Myrmecophaga tridactyla*), pampas deer (*Ozotoceros bezoarticus*), collared peccary (*Pecari tajacu),* tapirs (*Tapirus terrestris*) and green-winged macaws (*Ara chloropterus*). A jaguar (*Panthera onca*) breeding programme has also begun [50]. Restrictions on agricultural activities	successful reintroductions of large animal species have led to recovery of viable populations, and restrictions on agriculture have promoted recovery within remnant forest fragments. Recovery of resident populations of marsh deer (*Blastocerus dichotomus*), capybara (*Hydrochoerus hydrochaeris*) and other species

## 3. Results

Our proposed monitoring framework was applicable to measure rewilding progress across the three different restoration contexts. The resultant scores exhibit clear trends resulting from the set of restoration strategies used in the three case studies (figure 2), although caution should be used when inferring general conclusions. The overall rewilding score increased across all sites as a result of the rewilding initiatives. The species richness and viability of populations of large animals increased systematically since the beginning of the projects, which is consistent with the successful active reintroduction efforts and spontaneous recolonization of species across sites. In addition, human outputs from ecosystems either decreased or remained stable across all sites, including notable reductions in hunting and agricultural production in both Millingerwaard and Iberá since the projects started. Landscape connectivity barely changed since the beginning of the rewilding initiatives, as new human infrastructure was not built nor removed. Finally, fire regimes have become more natural over the course of the rewilding initiatives in areas where the fire is an important ecological driver either naturally or because of management.

While the rewilding scores increased over time in the different areas, the magnitude of changes in ecological integrity and human forcing differed across sites. The Millingerwaard project started from a considerably less wild baseline than the other projects, but it experienced substantial increases in the natural system's condition along both dimensions (figure 2). This improvement was in part associated with the transition from farmland to natural grazing areas and the restoration of the natural hydrological regime via dam and dyke removal. The Swiss National Park has undergone a complete reduction in direct human inputs and outputs since 1914, driven by the end of the Alpine ibex (*Capra ibex*) reintroduction programme occurring in the reserve's early years and accompanied by artificial feeding initiatives (figure 2). Over the course of the project, the ecological succession has significantly progressed in the area. Had it not been for the reservoirs that were built during this period within the park's boundaries, the ecological integrity score would have increased even more. This infrastructure fragmented the aquatic habitats and affected the natural hydrological regime, which is now artificially regulated to improve the ecology of the river [51]. Finally, the Iberá project has experienced an increase in ecological integrity over the past decades, mainly driven by increases in the number of large mammal species and the viability of populations associated with the project's ambitious reintroduction and population reinforcement programmes [50] and woody expansion (figure 2). On the other hand, the associated intensive management effort to facilitate the recovery of wildlife species that were hunted to extinction during the twentieth century has increased the human inputs in Iberá. Nevertheless, it is expected that this score will improve in future years if the reintroductions are successful and these management activities can be reduced.

## 4. Discussion

This is the first attempt at establishing and implementing a generalized practical rewilding monitoring framework, meeting a clear need highlighted in restoration [52]. Our study also fills an important gap in applied rewilding science related to the identification of a set of restoration actions and their associated results. Measuring rewilding progress facilitates the achievement of several goals, including (i) assessing changes in the ecological integrity of ecosystems and the reduction of human forcing over them and (ii) incentivizing rewilding ambitions beyond a single component of the framework. The multiplicative nature of our rewilding score, in contrast with an alternative additive approach, emphasizes the interactions between the different components. That is, the rewilding score is not a simple addition of its components, but results from their synergistic combination.

One strength of this framework is that it recognizes that reducing direct human inputs and outputs into the system might not immediately translate into an increase of the system's ecological integrity. Specifically, this may occur because of long lag times of recovery or land-use legacies in systems that have undergone intense landscape transformation resulting from intensive management or infrastructure development. An obvious example—which many rewilding projects address—is the large-scale extirpation of ecologically important species, where recovery may lie hundreds or thousands of years into the future without assistance [53]. Therefore, while the framework promotes initial interventions through immediate changes in the human forcing, it also lays out long-term ecological targets for the system (i.e. the recovery of more complex ecosystems), providing guiding goals for rewilding in the medium- and long-term. Moreover, the fact that the framework monitors not only the condition of the ecosystem at a given time, but also how human activities might be expected to influence its future condition, makes the framework a forward-looking approach for monitoring restoration outcomes.

The rewilding assessment framework provides readily applicable indicators to measure progress in projects involving very different spatial and temporal scales and under contrasting settings, from urban areas to extensive natural land. However, the framework also allows for the refinement and inclusion of new indicators as needed. Future iterations might incorporate the community composition of aquatic systems in a manner similar to the one we have implemented for terrestrial communities and potentially include indicators representing the degree to which large-bodied terrestrial and aquatic species are able to fulfil their ecological function. The framework could even be taken forward to marine ecosystems [52]. The addition of biodiversity indicators of small-bodied species such as insect community composition and diversity would assist with capturing rapid ecological changes resulting from restoration actions [54]. Information from the surrounding landscape could help identify off-site influences, which in some cases may need to be reduced or eliminated before restoration can be successful. For instance, expanding the connectivity indicators to capture regional-scale connectivity would facilitate understanding the role of the area in landscape-scale processes such as metapopulation dynamics, dispersal and migration [55]. Some authors have argued in favour of substitutions for restoring missing ecosystem functions [8,56]. Recognizing the uncertainties and controversies associated with these taxon substitutions [57], these could be eventually integrated into the framework for those cases where evidence-based guidelines for implementing taxon substitution become available. Finally, indicators that are currently qualitative because of lack of data availability, or the requirement of prohibitive technical skills, might be transformed into quantitative indicators when high-quality data are readily available.

While we contend that our approach reasonably captures rewilding progress, we acknowledge a set of limitations to be addressed in future work. Firstly, caution should be taken when comparing the progress of initiatives occurring over considerably different spatial or temporal scales. For example, the Millingerwaard project scored more positively on the connectivity indicator than the Swiss National Park project, despite the latter containing a far greater extent of continuous habitats owing to the project covering an area 20 times larger. Furthermore, the changes in ecological integrity in the Swiss National Park have occurred over the past century, in contrast with 28 and 19 years associated with the Millingerwaard and the Iberá projects, respectively. Comparisons of the absolute magnitude of the changes in *R* scores and its components between different sites should appreciate the alternative spatial and temporal contexts. It is particularly important to note that changes in the naturalness components of the score (*E*) are more likely to occur in the mid- to long-term.

Secondly, some of the indicators are more sensitive to changes than others, meaning that differential amounts of effort are required to induce changes in the various indicators. For instance, reducing agricultural production or removing a large dam requires more effort than ceasing deadwood removal. Future iterations of the framework might weight the different indicator contributions to the overall score relative to the sensitivity of those indicators [58]; this would prevent rewilding initiatives from ‘gaming’ their scores by selecting management actions that are easier to pursue without confronting some of the more critical constraints [59,60].

As for other types of restoration [23,24], the goals of rewilding projects go beyond promoting self-sustained ecosystems and their success depends on the local context and the way they benefit and engage with people [61]. Our method focuses on measuring human forcing on ecosystems and their ecological integrity, which may in some cases induce trade-offs between rewilding and alternative socio-economic objectives [62]. However, human activities are penalized depending on how they affect ecosystem processes, so sustainable uses with minimal impacts on ecological processes will have little impact on the rewilding score. In reality, all but the uppermost extreme system's scores can be achieved while balancing a multitude of socio-economic benefits [63].

The precise shape of the mathematical functions integrating the different sub-components and the components themselves into the rewilding score is also an area for further research. For instance, in order to capture that low human interventions can be acceptable in our framework, a nonlinear response for the human forcing could be used to convert the sum of the indicator scores into the aggregate score, instead of the arithmetic average we propose. Work with stakeholders could further elaborate on the shapes that better capture the expert assessment of rewilding progress in a range of scenarios.

We stress that achieving the highest score should not be considered as the default objective or ambition, but that gradual increases in the natural condition of ecosystems at lower and intermediate scores can constitute a sensitive restoration target in many situations where it is critical to balance the socio-economic consequences. In these cases, the rewilding assessment framework should be used in conjunction with other socio-economic management objectives to optimize the trade-off between maximizing ecosystem integrity and delivering sustainable socio-economic value to communities and users [64]. For instance, involving people through multiple avenues—from participation to sustainable consumption of ecosystem goods and services to cultural renewal—can promote public engagement and stewardship of local ecosystems and improve restoration success [65].

As a concluding remark, the rewilding assessment framework presented here responds to calls to better integrate the science and practice of rewilding [66,67]. Although there are challenges remaining, we believe that the implementation and further development of our monitoring framework will help catalyse a positive and ambitious vision for rewilding. Furthermore, the application of this framework provides guidance for practitioners, funders and decision-makers to incorporate or demand a multifaceted perspective for rewilding initiatives and, simultaneously, incentivize conservation initiatives to go beyond the recovery of species and habitats and include ecosystem function and processes.

## Supplementary Material

Supplementary tables

## Supplementary Material

Data

## Data accessibility

The datasets supporting this article have been uploaded as part of the electronic supplementary material.
