# Correction to ‘Measuring rewilding progress’

**DOI:** 10.1098/rstb.2018.0568

**Published:** 2018-12-17

**Authors:** Aurora Torres, Néstor Fernández, Sophus zu Ermgassen, Wouter Helmer, Eloy Revilla, Deli Saavedra, Andrea Perino, Anne Mimet, José M. Rey-Benayas, Nuria Selva, Frans Schepers, Jens-Christian Svenning, Henrique M. Pereira

*Phil. Trans. R. Soc. B* **373**, 20170433. (Published Online 22 October 2018). (doi:10.1098/rstb.2017.0433)

The author names in Reference 12 were incorrect. The reference should read as follows:

12. zu Ermgassen SOSE, McKenna T, Gordon J, Willcock S. 2018 Ecosystem service responses to rewilding: first-order estimates from 27 years of rewilding in the Scottish Highlands. *Int. J. Biodivers. Sci. Ecosyst. Serv. Manag.* **14**, 165–178. (doi:10.1080/21513732.2018.1502209)

