# Supplementary material for: Measuring rewilding progress
Source: Philos Trans R Soc Lond B Biol Sci. 2018 Oct 22;373(1761):20170433. doi: 10.1098/rstb.2017.0433 (PMC6231071; doi:10.1098/rstb.2017.0433)
Supplement: Supplementary tables [file rstb20170433supp1.pdf]

Supplementary materials to ‘Measuring rewilding progress’

A ‘practitioners brief’ with detailed description of the indicators from the proposed framework including scoring, how to calculate them and which data use is available in **table S1**.

Additional information on the summarized evidence collected for the effectiveness of restoration actions tied to indicators of the framework is available in **table S2**.

Table S1. Practitioners guide to measuring and evaluating each indicator within the rewilding assessment framework.

Pressure and State variables	Description	Score	How to measure	Examples
**DIRECT HUMAN INPUTS AND OUTPUTS**				
Artificial feeding of wildlife	Is artificial feeding of large animals allowed, and does it occur in the study system? Artificial feeding might come in the form of: maximizing the physical condition or the survival of individuals of game species; supporting species populations of conservation interest during food shortage periods; attracting individuals to specific points to e.g. facilitate hunting or wildlife sighting; or as a diversionary measure to reduce wildlife damages. To what extent may be the ecosystem affected by artificial feeding? If this activity was banned, how strongly would the system change?	0 - No artificial feeding;<br>0.5 - Some type of artificial feeding is provided at levels unlikely to significantly affect animal movements and other ecological processes;<br>1 - High levels of artificial feeding and/or evidence for supplementary feeding affecting ecological processes	When judging the impact of supplementary feeding on the study system here, take two major factors into account: the **intensity** of feeding in the study system, and the **total area** within the system affected. More **intense** feeding regimes are those that experts and ecological managers notice they greatly affect the behaviour of the animals in the system, such as by creating a honey-pot effect where many of the different large-bodied species in the system come to feed regularly or daily. In intense systems, food might be provided daily or very frequently, and the amount of food provided would be enough to sustain large groups of animals. Less intense feeding regimes are those that are infrequent, and the amount of food provided is only enough to support a few animals, such as when a household throws its food waste outside to allow wild animals to feed on them. If there are many sources of supplementary feeding throughout the landscape, or a few very intense feeding stations, then the area affected by supplementary feeding is likely to be large.	0.2 - In the landscape there are a few feeding stations that are specifically targeted at supporting vulnerable lynxes, and lynxes alone, so they can survive the winter.<br>0.4 - In a large rewilded area, a few of the hotels use their food waste to attract wildlife to certain viewing areas. They do this once a week, and the amount of food thrown out is enough to attract a few animals.<br>0.6 - In a large rewilding area, there is one large vulture feeding point used to attract tourists. It attracts many animals from the local region, but the majority of the rewilding area is unaffected.<br>1 - In a small rewilding area, there is one large feeding station that all of the animals throughout the area are attracted to daily. It is their main source of food.
Population reinforcement	Have animals (scavengers, large herbivores, carnivores and their associated important prey species) been anthropogenically (re-) introduced into the system	0 - No population reinforcement at least during the last year;<br>0.5 - Species populations of conservation concern	The reinforcement or reintroduction of a species can be judged to be intensive if it is a key factor in preventing the species from becoming locally extinct, probably because the species local mortality rate is high or because reproductive success in the species locally is very low. Less intense reintroductions or population	0.5 - A family of a rare species that was previously locally extinct is reintroduced into the rewilding area as a first attempt at permanent reintroduction.

	in the last year? Are managers constantly / regularly (i.e., every year) reinforcing wildlife populations by bringing individuals in from other populations?	sporadically reinforced to improve their conservation status<br>1 - Regular to intensive population reinforcement for the conservation of populations that would otherwise decline, or reinforcement of non-declining populations or populations of no conservation concern.	reinforcements can be considered as those that are either a) not essential for the species' local survival, but instead to e.g. increase the probability of an individual meeting a mate or to promote genetic diversity; or b) a small population of a species is reintroduced to see if it can persist and without the intention of following this reintroduction up with a larger reintroduction.	0.5 - A small wildcat population is in danger from reduced genetic diversity. A couple of individuals from another population are introduced to boost local genetic diversity.<br>1 - There is a high mortality rate for one of the species in the rewilding area; so, while the cause is investigated, new individuals are introduced into the system every year to keep the population stable.
Agricultural outputs	How intense is the arable (crops) farming in the area? Is the farming applied in the rewilding area extensive and suitable for a wide variety of wildlife, or highly intensive commercial agriculture with very few species persisting on their fields? Have farms in the area been abandoned?	$\sum H_{crop} \times \%_{crop}$<br>Where:<br>$\%_{crop}$ = proportion of the total rewilding area devoted to cropland,<br>H_{crop} =<br>0 - No harvested or fallow for at least 5 years (i.e. land abandonment);<br>0.5 - Cropped and harvested under traditional, extensive farming practices;<br>1 - Intensive farming	Consider all of the cropland in the rewilding area. Firstly, how much of the rewilding area is devoted to crops (by percentage area or total area)? Secondly, what is your best judgement of the proportions of this cropland devoted to intensive vs extensive farming vs abandoned land? Fallow (i.e. fields that are being set aside in order to regenerate with the intention of using them in future harvests) must have been unused for at least 5 years to count as abandoned. Intensive farming implies a high use of fertilizers, pesticides, and machinery.	0.2 - 20% of the rewilding area is devoted to intensive commercial agriculture.<br>0.2- 40% of the rewilding area is devoted to crops using traditional farming practices.
Forestry outputs	How much of the forest in the rewilding area is devoted to commercial forestry, and how responsible are the harvesting practices of the main plantations?	$\sum H_{logg} \times \%_{logg}$<br>Where:<br>$\%_{logg}$ = proportion of the total rewilding area	Consider all of the forest in the rewilding area. Firstly, how much of the rewilding area is forested? Secondly, what is your best judgement of the proportions of this forest devoted to forestry that employs clear-cut logging vs selective logging of only high-value trees?	0.2 - 20% of the rewilding area is devoted to forestry that employs clear-cutting.<br>0.25 - 50% of the rewilding area is forested, and selective logging is employed throughout.

		devoted to production forestry,<br>H_{logg} =<br>0 - No logging;<br>0.5 - Selective logging;<br>1 - Clear-cut logging		
Grasslands outputs	How intense is the pastoral (livestock and hay) farming in the area? Is the farming extensive or highly intensive? Have farms in the area been abandoned?	$\sum H_{grass} \times \%_{grass}$<br>Where:<br>$\%_{grass}$ = proportion of total rewilding area devoted to intensively used grasslands,<br>H_{grass} =<br>0 - No harvesting for at least 5 years (land abandonment);<br>0.5 - Mowed or grazed under traditional, extensive farming practices;<br>1 - Intensive harvesting or high densities of commercial livestock	Consider all of the livestock farming in the rewilding area. Firstly, how much of the rewilding area is devoted to livestock (by percentage area or total area)? Secondly, what is your best judgement of the proportions of this livestock agriculture devoted to intensive vs extensive farming vs abandoned land? Extensive livestock farming might take the form of low densities of livestock living within wood pastures where other pasture-affiliated species can persist. Intensive livestock farming would be pasture with high stocking densities where there is little environmental heterogeneity. Fallow (i.e. fields that are being set aside in order to regenerate with the intention of using them in future harvests) must have been unused for at least 5 years to count as abandoned.<br>Free-roaming wild ungulates do not count towards this indicator, as they are considered a natural component of the system and not an 'output'.	0.2 - 20% of the rewilding area is intensive pastures with high densities of livestock.<br>0.4 - 80% of the rewilding area is wood pasture containing low densities of wide-ranging livestock.
Mining outputs	How intense are the ecosystem impacts of mining in this area, and what is the total area affected?	$\sum H_{mine} \times \%_{mine}$<br>Where:<br>$\%_{mine}$ = proportion of total rewilding area devoted to open mining,<br>H_{mine} =<br>0 - No mining;	Consider the percentage of the total land in the rewilding area that is open mines. How intense is the mining employed in this region? Is it large-scale and a cause of widespread ecological impacts, or more localised and with few wider ecological impacts?	0.05 - 5% of the rewilding area is open-cast mining, and mining operations are known to increase pollution levels in local water supplies.<br>0.025 - 5% of the rewilding area is a mining site, but mining has limited ecological impacts.

		0.5 - Mining with non-destructive production practices and strict regulation of pollution;<br>1 - Intensive mining with destructive mining practices and clear evidence of pollution		
Harvesting of terrestrial wildlife	Is hunting allowed? To what extent is the ecosystem affected by hunting? If this activity was banned, how strongly would it affect the target species and others with which they interact?	0 - No hunting;<br>0.5 - Low levels of hunting unlikely to significantly affect the growth rates of wildlife populations, animal movements, or species with which hunted species interact;<br>1 - High levels of hunting and/or probable or demonstrated effects on the growth rates and/or the population structure of harvested populations or species interactions	The hunting score here should be given considering whether it is sustainable (i.e. it does not impact the ability of the hunted population to self-regulate); and whether it affects species that are present in lower densities than one would expect in natural systems. Selective trophy or game hunting of ungulates in a system where ungulate populations are otherwise unregulated because there is an absence of predators to control their populations would therefore be considered less intense, as would the hunting of invasive species, and hunting of top predators in a system where they are already at low densities because of risks of predator - livestock conflict would therefore be considered more intense.	0.2 - Selective and highly controlled hunting of deer in a rewilding area where there are no top predators.<br>1- Hunting of wolves in an area with high ungulate populations which are increasing because of a lack of predators.
Harvesting of aquatic wildlife	Is extractive fishing allowed? To what extent is the ecosystem affected by extractive fishing? If this activity was banned, how strongly would it affect the target species and others with which they interact?	0 - No extractive fishing;<br>0.5 - Low levels of extractive fishing unlikely to significantly affect the growth rates of wildlife populations, animal movements, or species with which fished species interact;	The fishing score here should be given considering whether it is sustainable (i.e. does not impact the ability of the fished population to self-regulate); and whether it affects species that are present in lower densities than one would expect in natural systems. Small-scale recreational fishing of species that are abundant in the system would therefore be considered less intense, whilst fishing for large, predatory fish that are known to be experiencing population declines would be considered more intense. Fishing in artificial ponds specifically designed	0.2 - In a large rewilding area, a small section of the river is occasionally visited by recreational fisherman catching and re-releasing common fish.<br>1- A commercial or intense recreational fishery operates in the rewilding area, and fishes large-bodied species that are known to be experiencing population declines in the area.

		1 - High levels of extractive fishing and/or probable or demonstrated effects on the growth rates and/or the population structure of harvested populations or species interactions	for fishing would also not be considered to have an intense impact on the ecosystem.	
Carrion removal	Is it permitted to leave large carcasses in the field according to regulation?	0 - Carcasses are left in the field;<br>1 - Carcasses are removed from the field	Carrion supply is a natural, unpredictable and pulsed resource that is sometimes removed for various purposes (e.g., disease control). What is the public / animal health legislation that affects whether or not carcasses are considered a health risk in the area? Does it require that carcasses are removed when they appear?	0 - Legislation in the rewilding area does not determine that carcasses must be removed from the field, and they are commonly left for scavengers.<br>1- Legislation in the rewilding area determines that carcasses must be removed for public / livestock health reasons.
Deadwood removal	Is deadwood (dead trees and woody debris) removed from within the rewilding area?	0 - No deadwood removal; 0.5 - Low levels of dead removal (e.g., roads and footpaths, and parks) unlikely to affect disturbance regime, animal movements and other ecological processes significantly; 1 - High levels of deadwood removal	Removing dead wood is a common management practice that can be perceived to improve the aesthetics of systems, however, dead wood can be a valuable habitat for numerous taxa. Removing dead wood only from areas where it is a danger to people or obstructs footpaths would be considered less intense, and removing dead wood from throughout forests in order to promote its aesthetics would be considered more intense.	0.1 – Dead wood removed from footpaths when trees fall and obstruct the way, but otherwise untouched<br>0.5 – all dead wood is removed from a wood pasture or park that is used by people in a small area within the rewilding area, but is otherwise left alone<br>1 – all dead wood is removed throughout the area for aesthetic purposes
**ECOLOGICAL INTEGRITY INDICATORS**				
**Disturbance regimes**				
Natural avalanche and/or rock slide regimes	Are there avalanche or rock slide avoidance measures in place (e.g., artificial slope bombardment, barriers) to	0 - Regulation of avalanches / rock slides across the whole rewilding area;	Do avalanches or rock slides occur in the rewilding area? If so, are there any human settlements / areas of high human activity that are potentially threatened by avalanches? In those areas, are the avalanche / rock slide	0.5 - In a mountainous rewilding area, slopes popular for skiing that cover a small amount of the rewilding area (e.g. <10%) are bombed to instigate controlled

	reduce their risk? Is the land-slip disturbance regime regulated?	0.5 - Regulation of avalanches / rock slides only in certain places;<br>1 - No regulation of the avalanche / rock slide regime	regimes controlled (e.g. through slope bombing to trigger controlled avalanches)? If those areas extend across the majority of the rewilding area, then the control of regimes can be considered to be high. If, however, there are large portions of the rewilding area where avalanches / rock slides are uncontrolled, the regulation of the avalanche regime can be considered to be low.	avalanches. Avalanches elsewhere are uncontrolled.
Natural fire regimes	Are there deviations of the natural fire regime due to human pressures (this might be in either direction ie. fire suppression, or prescribed burning)?	0 - Fire regime is heavily modified by human intervention including both artificial burning and/or fire suppression;<br>0.5 - Artificial burning and/or fire suppression is very localized and only cause minor ecological impacts;<br>1 - There are no deviations of the natural fire regime	Consider whether fires commonly occur in the rewilding area. The judgement regarding whether the human influence over fire regimes is intense should be influenced by two main factors: 1) are fires managed in the rewilding area, either through suppressing them when they occur, or through using fire as a conservation tool to restore soils and regulate vegetation?; and 2) is the vegetation in the rewilding area highly modified by people and the way that people have used the landscape in the past, and does this modified vegetation regime alter the intensity or frequency of fires in the system from what you would expect if intact, unmodified vegetation communities were in place? Deviations of the fire regime would be considered intense if prescribed burning is used as a conservation tool to maintain a certain ecosystem (e.g. moorland which would otherwise be forest), or if, as a result of human influences, a landscape that would previously have been covered by forest that underwent periodic natural forest fires is now covered by grassland where fires rarely occur	0 - A rewilding area that was pine woodland in the distant past that was adapted to occasional forest fires, and where fires played an important role in natural succession, is now an overgrazed pastureland, and no natural fires occur anymore because fuel load is insufficient.<br>0.5 - In a large rewilding area, a small section of it (e.g. <10%) is managed to promote moorland. Prescribed burning is used in this area, but a natural fire regime exists in the rest of the rewilding area.
Natural hydrological regimes	Are there measures in place (e.g., dams, dykes) to alter natural hydrological regimes and the risk of flooding?	0 - High regulation of the hydrological regime;<br>0.5 - Dams in place, but cause only minor impacts on the overall flood regime;	When judging the hydrological regime score to assign to the area, consider the total area of the rewilding site or region that is susceptible to flooding and/or where there are natural floodplains that experience regular flooding. If no flooding occurs, is it because man-made structures prevent flooding and regulate the	0 - Nearly all of the floodplains in the rewilding area never flood, or only flood catastrophically when man-made flood prevention infrastructure fails.

		1 - No regulation of the hydrological regime	river's flow? Flood modification should be considered more intense if a large proportion of the flood plains in the rewilding region are prevented from flooding by man-made barriers and modifications. Flooding should be considered less intense if a few of the waterways in the rewilding area are dammed, but the majority of the flood plains experience natural flooding regimes.	0.5 - In a large rewilding area spanning multiple flood plains, the hydrological regime on one of them is heavily regulated (i.e. no natural flooding occurs), but the flood regime on the others is unmodified.<br>0.5 - In a small rewilding area, a number of drainage channels have been built, so that when flooding occurs as normal, its intensity is lower, but it still floods the floodplain with a regular and natural frequency.
Natural pest regimes and mortality events	Are natural pest regimes and mortality events regulated? Are management actions implemented after mortality events (e.g., storms, pests)?	0 - No management to avoid pests or after mortality events;<br>0.5 - Low levels of management to avoid pests or after mortality events unlikely to affect disturbance regime, animal movements and other ecological processes significantly;<br>1 - Management actions implemented to avoid pests (e.g., pesticide use) or after mortality events (e.g., salvage logging, removal of burnt wood).	Natural pests, storms and other mortality factors may naturally occur in the system as a natural source of disturbance. This indicator would be considered less intense if highly localised or tree-specific pest control measures are used, such as individual tree chemical treatments, and more intense if pesticides are routinely used throughout the area.<br>If trees are treated for invasive species, this is exempt from this indicator as they are not considered ‘natural’ pests in the system.	0 – insecticides are routinely used throughout the rewilding area, either to protect crops or nuisance pests (eg. mosquitos)<br>0.7 – several individual high-value trees are treated with systemic pest control chemicals, but the majority of trees are untreated
**Landscape composition and connectivity**				
Terrestrial landscapes fragmentation	To what extent is the landscape fragmented by human infrastructure?	0 - Landscape is highly fragmented by humans	Landscape fragmentation and barriers that results from human infrastructures. Specifically, this metric is based on the probability that any	0.5 – a large road divides the rewilding area exactly in two

	What is the effective mesh size (m_{eff}) of the rewilding area?	(fully covered with infrastructure);<br>1 - Landscape is not fragmented by humans;	two points chosen randomly in an area are connected and are not separated by any barriers (the effective mesh size). Here the fragmentation geometry includes only human-made barriers (mainly roads and built-up areas).	
Aquatic landscapes fragmentation	What is the state of migratory paths in aquatic systems? To what extent are migratory processes in river systems allowed?	0 - Fish migration is highly regulated;<br>0.5 - Dams in place but alternative migration routes or fish cannons provided;<br>1 - No impediments to fish migration	Consider the waterways existing in the rewilding area. Are there multiple man-made elements that 'break-up' the waterways into different sections (e.g. dams), and do these barriers prevent fish from migrating up the waterways? Are there alternative routes that the fish could potentially use to reach upstream destination that have no barriers in them?	0 - Migratory species are unable to move upstream.<br>0.5 - There are multiple dams segmenting the main rivers in the rewilding area, but fish cannons have been installed so that fish can successfully overcome these barriers.<br>0.5 - One large dam prevents fish from migrating up one section of the river system, but there are alternative routes up the system which have no infrastructure in them blocking the way, and there is evidence that the migratory species use these alternative routes.
Spontaneous vegetation dynamics	What is the state of ecological succession throughout the rewilding area? How long have different areas within the area been since major human impacts?	$\sum H_{time\ since\ abandonme}$ $\times\ \%_{svd}$<br>Where: $\%_{svd}$ = proportion of area where spontaneous vegetation dynamics are allowed,<br>$H_{time\ since\ abandonment}$ = 0.1 – early successional stages (< 50 years); 0.5 - (50 – 200 years); 1 - last successional stages adapted to each ecological region or	Consider the percentage of the total land in the rewilding area that is habitat of varying successional states. How long since these areas were heavily disturbed by anthropogenic pressures? If the system is one where rates of natural disturbance are high (e.g., flood plain), then early successional states can still score highly if they have not been modified by people for an equivalent amount of time.	0.55 – Half of the rewilding area is recently abandoned land, and the other half intact old-growth forest >200 years old

		biome (e.g., >200 years old growth forest), or an equivalent number of years since anthropogenic disturbances in systems with high rates of natural disturbance		
Harmful invasive species	What is the impact of harmful invasive species on the rewilding area? Do they significantly impact natural processes or ecological communities? If the invasive species were removed, how would the ecosystem change in response to the relaxation of invasive pressure?	0 - Very severe impacts of invasive species on ecological communities in rewilding area;<br>0.5 - Impacts of invasive species within small, localised communities within rewilding area;<br>1 - No major invasive species present	Are there any major invasive species in the rewilding area that are having significant qualitative impacts on the existing vegetation or faunal community? Are these invasives concentrated into specific areas in the ecosystem, or have they successfully spread across the entire ecosystem? If the invasives are having a strong qualitative impact on the community across large sections of the rewilding area, this indicator would be given a low score. If invasive species are co-existing with native species without outcompeting them, or are localised to specific small areas, then a high score would be given.	0 – An invasive plant species (eg. blackberry) dominates the entire ecosystem, outcompeting native shrubs across large spatial extents<br>0.5 – An invasive plant species (eg. Japanese knotweed) dominates in one lake in the rewilding area, but is not found in other areas<br>0.8 – a non-native plant is found throughout the rewilding area, but only at low densities and without threatening local diversity. There is no qualitative impact on ecosystem processes
**Trophic processes**				
Terrestrial large-bodied fauna (>5 kg)	Species composition of large-bodied (>5kg) species relative to the maximum natural number of species and their maximum natural densities	$\frac{\sum S_{curr} * T_{curr} * V_{curr}}{\sum S_{max} * T_{max} * V_{max}}$<br>Where *S* is the space occupied by the species in the area, estimated from 0-1;<br>*T* is the percentage of the time in a year that the species is present in the area it occupies, estimated from 0-1;	For the top half of the fraction, consider one-by-one all of the large (>5 kg) species present in the rewilding area. What percentage of the suitable habitat within the overall rewilding area do you judge to be occupied by this species? If these species are highly mobile or/and population sizes are small, what percentage of the year does this species reside in the rewilding area? What is the viability of the local population of that species that exists within or around the rewilding area? A species should be considered of low viability if only a couple of individuals exist in the landscape and if either	0.2525 – Today, we have 2 large animal species in the rewilding area, and one of them is present throughout the whole area all year round. This species would score 1*1*1=1. The second species is a very small population that only exists in a small area on one edge of the rewilding area. It migrates in and out of the park, and so is only present in the park 3 months per year. It is from aa

	V is the viability of the population to which the individuals of the species belong, estimated from 0-1. This viable population might extend beyond the rewilding area (eg. if the species have large ranges), but as long as the complete local population is viable, this will score 1<br>*curr* denotes the current values for species in the system at the chosen point in time<br>and *max* denotes the maximum possible score for that species, which is always 1	the population would be seriously harmed if one or a few individuals would die or the reproductive success of the population is very low so the population is declining over time. For each species, multiply these 3 scores together to get an overall score for the species (which can be thought of as a proxy for this species' ability to carry out its ecological function in the rewilding area), then sum the scores for all species together. Note that migratory species should not be given a low score for time spent by default, instead they should be given a score of 1 if they appear in the rewilding area as part of their migration.<br>The bottom half of the fraction is the reference value, i.e. what is the maximum number of naturally occurring species that we could feasibly expect to occur in the system today? We then assume that all of these populations would be carrying out their ecological function completely.	local population that is highly threatened, and therefore its viability may be low. This species might score 0.2*0.25*0.2=0.01. Then, to get the score for the species composition at this time, we sum 1+0.01=1.01.<br>The aim of the rewilding project is to reintroduce a further 2 large animal species back to the rewilding area. Therefore, the reference value on the bottom of the fraction would be 4 species all completely fulfilling their ecological function, 1+1+1+1=4.<br>The overall species composition score therefore for the chosen time period is 1.01/4=0.2525.

Table S2. Summary of the evidence base for proposed restoration actions. EF=Effectiveness of restoration actions at achieving rewilding objectives (restoring trophic processes, landscape connectivity and composition, natural disturbance regimes and/or biodiversity). EF=0: No evidence or unknown effectiveness of restoration action; EF=1: Likely to be ineffective; EF=2: Trade-off between benefit and harm; EF=3: Likely to be beneficial; EF=4: Beneficial.

**Restoration action**	**List of references**	**EF**
Minimise artificial feeding of wildlife<br><br>There are multiple ecosystem impacts of supplementary feeding, most of which are likely to indirectly impact the system's trophic processes, disturbance and connectivity. In relaxing food constraints, feeding can promote survival during bottleneck periods (e.g. overwinter survival in the arctic fox [1]), and reverse the decline of threatened species populations limited by prey abundance (eg Iberian lynx [2]). However, feeding also introduces other risks to animal populations, including for example an increased risk of disease transmission caused by the dense spatial aggregation of animals at feed stations [3,4]. Feeding may also influence trophic processes by reducing predator requirements to naturally hunt prey: there is evidence in raptors that artificial feeding may lead to reductions in natural predation [5]. However, there is to the best of our knowledge no evidence in carnivorous mammals that artificial feeding reduces predation rates on natural prey. Artificial feeding is unlikely to interfere significantly with natural disturbances, with the exception of potentially intensifying herbivory or predation effects in the spatial vicinity of feeding stations as a result of enhanced herbivore or predator densities [6,7]. There is strong evidence that artificial feeding triggers changes in the ranges and spatial behaviour of large mammals (reviewed in [8]), including recorded examples of range contraction and reduction in animal movements (including migration) in response to artificial feeding [9,10].	[1], [2], [3], [4], [5], [6], [7], [8], [9], [10]	2
Minimise anthropogenic reinforcement of animal populations<br><br>Whilst the reintroduction and/or reinforcement of animal populations is justified from a conservation perspective in the case of local extinctions where the fundamental driver has been addressed [11], low genetic diversity that threatens population viability [12], or supplementing small populations to increase their growth rate [11], rewilding seeks to reduce anthropogenic augmentation of wildlife populations in the long-term following the recovery of self-sustaining populations. Aside from frequent population supplementation being expensive [13], the interactions of multiple self-sustaining populations of species is fundamental to a system's ecology, and an important component of ecosystem stability [14], diversity [15,16], and evolution [17]. We note, however, that even population reintroductions that are deemed successful at restoring self-sustaining populations at any given time require constant monitoring and potentially further intervention if unexpected declines occur [18].	[11], [12], [13], [14], [15], [16], [17], [18]	0
Reduce hunting of terrestrial wildlife<br><br>As one of the dominant threats to large animals globally [19,20], hunting has both direct and indirect impacts on populations of game species, affecting their overall population sizes, behaviour, and thus ecological function. In European systems, it is estimated that predator densities would be significantly higher if prey-limited [21], thus implying that top-down effects such as hunting are constraining predators' function and disrupting trophic relationships [22]. This has the potential to initiate cascading effects throughout ecosystems, such as promoting increases in ungulate abundances and its associated ecological effects such as the alteration of plant and animal communities and changes in the nutrient cycling [23]. Hunting also causes demographic effects on large animal populations, as it generally targets individuals of larger body size and higher trophy value, potentially leading to skewed sex ratios and the disruption of dominance hierarchies with associated effects on population dynamics and demography [24,25]. Furthermore, hunting alters the spatio-temporal distribution of predation and herbivory across landscapes through human mediated behavioural effects [26,27], further affecting trophic and evolutionary processes at landscape scales. In affecting herbivore spatial behaviour, hunting has the potential to alter natural disturbance regimes by directing herbivory towards locations where	[19], [20], [21], [22], [23], [24], [25], [26], [27], [28]	4

the perceived threat level is lower [26]. Finally, hunting at species range margins might interact with other factors to inhibit range expansion and population connectivity [28].		
Reduce extractive fishing<br><br>Fishing and recreational angling systematically remove specific, higher-value species and especially larger size-classes from aquatic systems, and thus have a corresponding impact on the trophic relationships within aquatic food webs [29]. Increased fishing pressure on individuals with large body sizes may trigger demographic effects within fish populations that can undermine stock resilience, such as causing lower juvenile survival as the most fecund potential parents are removed from the breeding pool [30]. Whilst in extreme cases recreational fishing can lead to population collapse (e.g. [31]), it is more likely to induce changes in community composition, risking triggering trophic cascades and loss of ecosystem function [29,32]. Extractive and recreational fishing also has ecological impacts outside the aquatic system - anglers can be a major cause of disturbance to riparian corridors, and can trigger anti-predator behaviours in wildlife that also have the potential to alter spatial trophic processes [29]. We found no evidence whether extractive fishing has major effects on ecosystem connectivity or interferes with natural disturbances.	[29], [30], [31], [32]	4
Allow carrion persistence in ecosystems<br><br>In converting an unpredictable, pulsed resource like wild carrion into a predictable and constant one, supplementary feeding has important ecological impacts on scavengers which are adapted to spatially and temporally unpredictable food sources (reviewed in [33]). On one hand, providing artificial feeding instead of wild carrion has been demonstrated to improve survival and abundances of some threatened species (eg Egyptian vultures [34]), and artificial feeding might be necessary if local carcasses have been poisoned [35]. On the other hand, carrion persistence has numerous positive ecological impacts potentially indirectly affecting trophic processes and connectivity. Predictable resources tend to attract larger groups of scavengers, facilitating increased intra-guild competition which tends to favour larger, more common species over smaller specialists [36], and thus carrion persistence counteracts this process and can release specialists from competition. Furthermore, increased animal densities around predictable food sources have been demonstrated to locally increase predation [37], potentially impacting on local trophic relationships. Unpredictable food sources are not associated with these impacts. Furthermore, providing pulsed, natural food sources facilitates natural scavenger spatial behaviour, whilst predictable sources alter spatial behaviour, with for example vultures preferentially choosing ranges encompassing feeding stations [38], which may impact on sub-population connectivity (although this has not been empirically evaluated). An important number of insects and decomposers, as well as facultative scavengers under harsh environmental conditions, are dependent on carrion[39,40].	[33], 34[], [35], [36], [37], [38], [39], [40]	2
Do not remove dead-wood in ecosystems<br><br>Evidence suggests that dead wood promotes biodiversity in forests [41,42], but that the positive impact on saproxylic taxa is significantly higher than that on non-saproxylic taxa [42]. Furthermore, the magnitude of the impacts on saproxylic taxa is dependent on contextual factors such as biome [41]. Dead wood has a variable impact on habitat connectivity, with some studies demonstrating positive associations between deadwood density and species ranges in saproxylic species (resulting from increased connectivity of appropriate habitat) [43], and others finding no effect [44]. The effect of deadwood on habitat connectivity for non-saproxylic or vertebrate taxa has to the best of our knowledge not been assessed. Dead wood has the potential to increase forest vulnerability to disturbances such as fire or pest outbreaks [45].	[41], [42], [43], [44], [45]	3
Reduce agricultural outputs - use abandoned land for wildlife<br><br>The abandonment of agricultural land leads to the creation of unmanaged land in which natural processes determine the rate and trajectory of succession. However, the direction of succession is heavily determined by the species community and ecosystem processes operating at the time	[46], [47], [48], [49], [50], [51], [52]	2

when the land first becomes abandoned; thus the impacts of land abandonment are heterogeneous and vary with landscape history [46,47]. In some contexts, land abandonment benefits biodiversity, but in others particularly with a long history of human land use abandonment can reduce species richness [47,48]. Abandoned land with low human presence has the potential to enhance trophic relationships by creating new habitat suitable for large carnivores [49], and thus increasing the habitat connectivity at landscape scales [50]. Abandonment may have varied effects on disturbance processes. In systems where disturbances are mainly anthropogenic, abandonment may reduce natural disturbance and therefore threaten species dependent on early successional states (eg [51]). However, abandonment may also increase rates of natural disturbances, by for example increasing fire fuel load and therefore accelerating the frequency of wildfires [52].		
Reduce forest product extraction<br><br>Extractive forestry and timber harvesting have a profound impact on forest ecosystems. Logging and selective logging of natural forests cause declines in forest-affiliated biodiversity, ecosystem resilience and services [53], and species richness is in general lower in managed than unmanaged forests [54]. Forest clearance causes wide-ranging changes in ecosystem function (eg [55]). Timber extraction of any kind causes forest fragmentation, which when coupled with reductions in habitat extent is associated with considerable impacts on biodiversity, especially forest specialist species [56,57]. These large associated impacts on ecological communities also spill over into trophic networks, with several top predators strongly associated with forest and deterred by forest fragmentation and contact with humans (eg lynx [58,59]). Timber removal and fragmentation associated with habitat loss inhibit ecosystem connectivity, potentially limiting species dispersal across landscapes and therefore increasing their vulnerability to environmental and anthropogenic pressures (eg [60,61]).	[53], [54], [55], [56], [57], [58], [59], [60], [61]	4
Encourage natural avalanche regimes<br><br>In much the same way as natural fire regimes (see below), avalanche regimes can play an important role in structuring ecosystems and initiating success that creates heterogeneous landscapes with opportunities for promoting biodiversity overall [62]. Avalanche frequency can be a driver of ecosystem structure, with systems with intensive avalanche regimes usually dominated by short, slow-growing trees relative to undisturbed areas [63]. Avalanches influence succession by reorganising the surviving vegetation and creating canopy openness, enhancing the growth of young surviving trees and creating structurally heterogeneous ecosystems which can promote species diversity [64]. Furthermore, the more open habitats created by avalanches are the favoured habitats of numerous large mammal species, including chamois [65], thus potentially spatially structuring trophic relationships.	[62], [63], [64], [65]	3
Encourage natural fire regimes<br><br>In systems with a long history of fire-related disturbances, multiple evolutionary adaptations have emerged to allow species to survive and achieve competitive advantage through fire-tolerance. In such systems, fire is a key ecosystem process that distributes succession in a random way across the landscape, creating a dynamic matrix of different age patches and improving habitat suitability for a wider range of species [66,67]. In fire-prone ecosystems that also contain large free-roaming herbivores, the interactions between fire disturbances and herbivory are key processes in organising ecosystem biodiversity, the spatio-temporal distribution of food web components, and ecosystem function [66,68]. For example, in US prairie ecosystems, fires induce increased aboveground biomass in burned patches, thus attracting grazers. This inhibits herbivory in unburned patches, facilitating succession and the accumulation of aboveground biomass, that both provides suitable habitat for less disturbance-adapted species, and increases fire risk to help re-initiate the spatio-temporal cycle of disturbance [66]. Multiple other examples of intimate ecological relationships between trophic processes and fire regimes exist, further demonstrating the direct and indirect role of fire in structuring food webs and spatio-temporal disturbance processes [68]. However, the effects of fire are context-specific, and careful consideration of the ecosystem's evolutionary history	[66], [67], [68], [69], [70], [71], [72]	3

should be taken before utilising fire as a management practice. Humans also commonly manipulate fire regimes by causing deliberate fires, either for agricultural or conservation management purposes. This can have large negative impacts on biodiversity and be a key driver of habitat loss, thus potentially reducing functional connectivity [69,70]. In these cases, reducing fire frequency can be an important strategy. In many systems, fire can facilitate invasion and undermine ecosystem service delivery [71,72].		
Restore natural hydrological regimes<br><br>Natural hydrological regimes have a profound and multidimensional impact on floodplain ecology [73]. In well-functioning floodplain systems, flood pulses can be associated with 'booms' in ecosystem productivity and food provision that influence the trophic relationships both within the aquatic system and the surrounding terrestrial landscape [74,75]. Flooding induces heterogeneous effects on biodiversity, generating both 'winner' and 'loser' species (eg [76]). Inundation events promote connectivity between multiple floodplain habitats, redistributing food between habitats and inducing dietary shifts that promote large-bodied fish species [77], thus influencing aquatic trophic networks. Similarly, flooding events influence terrestrial trophic networks, but these linkages between terrestrial and aquatic systems remain understudied [78]. Floods are important disturbance events, that can help reset successional processes and hence generate a spatially- and temporally-changing habitat mosaic that can provide diverse opportunities for species with a range of disturbance-tolerances [79,80], although it should be noted that by connecting spatially-separated floodplain micro-communities floods tend to cause community homogenisation in the short term [81]. In some cases, floods can stimulate plant recruitment by re-setting succession [82], potentially benefitting species that utilise dense riverine vegetation [83]. However, although the evidence that altering hydrological regimes has negative ecological consequences on floodplain systems is strong, much less research has been conducted on modified floodplain responses to the re-instatement of hydrological regimes. Preliminary evidence does indicate that floodplain restoration can lead to the recovery of at least some aspects of the ecological community present before hydrological regime modification [84,85].	[73], [74], [75], [76], [77], [78], [79], [80], [81], [82], [83], [84], [85]	3
Allow natural pest and mortality regimes<br><br>Pests and diseases can play important roles in structuring ecosystems and influencing succession [86,87]. Wildlife diseases can affect trophic processes by altering the relative abundance of important species in the trophic network, triggering cascading effects [86]. For example, facial tumour disease has had large impacts on the mortality of Tasmanian devils, reducing their influence on ecosystem processes and potentially facilitating mesopredator release [88]. Plant pathogens can create disturbances that mimic successional processes, such as causing canopy die-off, creating opportunities for early-successional species [87]. Such disturbances create a range of winner and loser species, with unpredictable impacts on biodiversity, which can include promoting species diversity [89].	[86], [87], [88], [89]	2
Removal of dams - reducing aquatic landscape fragmentation<br><br>Dams disrupt downstream hydrological flow regimes, with wide-ranging ecological consequences [90], including a general community shift towards species adapted to stable flows [83]. This community shift is naturally associated with a large change in riverine trophic relationships [91]. Furthermore, dams interrupt the connectivity of river systems and reduce downstream natural hydrological regimes and their associated ecological disturbances [83]. Removing dams can therefore help restore these processes, although after prolonged ecosystem change the community post-dam removal is unlikely to be identical to that pre-dam construction [92]. Restoring dams has been demonstrated to restore ecological connectivity [93] (although this can also increase the transmission of invasive species [94]), and also to help increase the rates of downstream natural disturbance events [82].	[82], [83], [90], [91], [92], [93], [94]	4

Reduce levels of human infrastructure - minimising terrestrial landscape fragmentation<br><br>The expanding footprint of human activities not only is causing the loss of habitats and biodiversity, but it is also affecting how animals move through fragmented and disturbed habitats. Animal movements are essential for ecosystem functioning because they act as mobile links [95] and mediate key processes such as seed dispersal, food web dynamics (including herbivory and predator-prey interactions), and metapopulation and disease dynamics [96], which as a whole have shown to increase ecosystem resilience by providing sources for reorganization after disturbance, i.e., ecological memory [95]. The density and distribution of human infrastructure has a significant impact on wildlife at regional scales [97,98], supported by more local studies which have shown the severe effects of reduced vagility on these processes [99,100]. At the global scale, Tucker et al. [101] found reduced vagility across mammalian species in relation with the human footprint, which suggests consequences for ecosystem functioning worldwide. Indeed, it is well established that dispersal between heterogeneous sites can influence patterns of diversity in metacommunities [102]. Furthermore, infrastructure can be particularly impactful on the range of large carnivores. For example, one study in Slovenia suggests that roads act as barriers to brown bear ranges if their traffic volumes are >5000 vehicles/day [103]. Examples of conservation interventions that have aimed to restore connectivity show that restoring connectivity can reduce population mortality and reconnect metacommunities [104]. On the other hand, increased connectivity can contribute to increasing the spread of invasive species and disease spread [105], but so can human infrastructure. Furthermore, whilst fragmentation is commonly associated with a reduction in habitat extent, which causes biodiversity loss through habitat loss [61,106], fragmentation per se has on average positive effects on biodiversity [56]. Based on our overall knowledge, landscape management should strive to maintain landscape permeability by enhancing animal movement as a key conservation target.	[56], [61], [95], [96], [97], [98], [99], [100], [101], [102], [103], [104], [105], [106]	4
Minimise harmful invasive species<br><br>Invasive species are one of the most important drivers of extinction globally, and can be key drivers behind ecosystem process alteration and loss [107,108]. Invasives can impact on natural disturbance regimes through multiple pathways. Firstly, invasives can alter fire frequency and severity, particularly through the establishment of invasive plants that can increase fuel load [108–110]. In some systems, this mechanism can increase fire frequency so severely that communities adapted to less intense fire regimes disappear completely and are replaced by assemblages of fire-tolerant species [109,110]. Secondly, invasive pest species can cause large-scale species-specific die-offs, which act as dramatic disturbance events and may result in significant shifts in community composition as unimpacted species exploit the newly-liberated ecological space [111,112]. Thirdly, there is evidence of invasive species altering hydrological regimes, although mostly in the form of altering the height of the water table rather than flood regimes [110]. Beyond influencing disturbance regimes, invasive species can also have significant direct impacts on trophic processes and community composition. Over time, the composition of impacted communities tends to shift towards species that are resistant to the characteristics of the invasive. For example, the introduction of invasive ungulates in Australia drove a shift towards browsing-tolerant species in the vegetation community [113]. Finally, invasives can alter the food web structure of ecosystems, triggering trophic cascades that further influence ecosystem processes [108]. For example, arctic fox (*Alopex* lagopus) introduction to the Aleutian archipelago led to seabird declines, impairing nutrient transport from sea to land and a change in community composition from grass- to shrub-dominated [114]. Whilst in theory removing invasives should ideally lead to the recovery of ecosystem processes, the elimination of established invasives is often prohibitively expensive and challenging [115,116].	[107], [108], [109], [110], [111], [112], [113], [114], [115], [116]	3
Recovery of large bodied species<br><br><u>Carnivores</u><br>It is now widely acknowledged that top predators have important ecological impacts on ecosystems by exerting direct and indirect top-down control on trophic networks [19,117] (but see [118]), with evidence suggesting that the magnitude of their impact is related to their body	[19], [20], [37], [48], [95], [117], [118], [119], [120], [121], [122], [123],	3

size [19]. The removal of top predators from trophic networks can trigger trophic cascades that may lead to significant loss of ecosystem function [117,119]. Thus, a key aim of rewilding is the recovery of large animals to restore the ecological trophic regulation [120]. This recovery can be promoted through different management actions, including the facilitation of natural recolonizations, the reinforcement of reduced populations, and the reintroduction of locally extinct species. As a result of the long timescales required to effectively evaluate the ecosystem impacts of large carnivores, information on the effectiveness of restoring trophic processes, connectivity and natural disturbances to areas where they were previously degraded is based on theory (often complimented by observing trophic cascades triggered by changing predator abundances within their native ecosystems) and relatively few reintroduction case studies. In theory, the recovery of top predator populations should exert top-down control on populations of mesopredators and prey species, leading to reductions in population size and changes in prey behaviour [121,122], thus altering their ecological role throughout the landscape. In many degraded systems where the past extirpation of predators has increased the ecological role of their prey and thus altered ecosystem function, the restoration of predators might be expected to help recover this function. In reintroducing the top connection in the trophic network, carnivore recovery necessarily increases ecosystem trophic complexity, and depending on the structure of the trophic network, this might trigger a wide range of ecological effects, from reducing herbivore browsing and promoting woody vegetation recovery, to changing disease dynamics and altering nutrient recycling (reviewed in [19]). Top predators may also facilitate complementary species such as scavengers [123], promoting biodiversity and expanding the abundance of complementary species within the trophic network. Beyond trophic processes, predators might also contribute to the restoration of ecological connectivity and promoting natural disturbance regimes. Predators can be important link species between multiple habitat types, enhancing the inter-habitat connectivity [95]. In marine systems, individual large predators have been shown to predate in multiple spatially separated habitat types, connecting the food webs of alternative habitats and potentially enhancing food web stability [124]. Predators may also alter natural disturbance regimes by mediating and altering the spatial pattern of herbivory of large herbivores [121].<br><br><u>Herbivores</u><br>Large herbivores have large ecological effects on ecosystems as a result of the ecological disturbance induced by their feeding patterns [20], which is subsequently likely to alter the relative species composition of ecosystems and affect trophic networks. They can also be important ecosystem engineers [125]. In general, large herbivores restrict woody plant growth and establishment except in inaccessible areas or where browsing is regulated by predation threat [126], driving a general shift in plant species composition towards defended browsing-tolerant species [126,127]. Heterogeneous grazing pressure is an important natural disturbance process that leads to spatially structured landscapes that may contain a mosaic of different habitat types in different successional stages [126], which may promote ecosystem biodiversity through diverse niche creation [48,128]. Large herbivores also play an important role in long-distance dispersal mechanisms, because of their large range sizes, and can thus connect plant habitat patches both within and between ecosystems [129]. As a result, the recovery of large herbivore ecological roles has been suggested as a potential mechanism for promoting ecosystem restoration in fragmented landscapes containing isolated degraded habitat patches [130].<br><br><u>Scavengers</u><br>Whilst the role of scavengers in several ecosystem processes such as nutrient cycling and disease control is widely acknowledged [131], their effect on trophic processes has received less attention. Most predators are scavengers to a certain extent. Scavengers interact with food web dynamics through multiple indirect pathways whose significance is only beginning to be explored [37,132]. Scavengers can induce higher predation rates in carnivores by reducing the availability of carrion and in certain contexts outcompeting carnivores at carrion sites [132,133]. Depending on the food-web structure, the reduction in carrion might induce bottom-up regulation of mesopredator populations, potentially reducing predation rates on smaller wildlife [134].	[124], [125], [126], [127], [128], [129], [130], [131], [132], [133], [134], [135], [136], [137]	

<u>Omnivores</u><br>Large-bodied omnivores have important ecological effects, both as an abundant prey source and as ecosystem engineers. In areas where their ranges overlap, omnivores such as wild boar can be the main source of prey for apex predators, partly because of their relative abundance, and partly because of their relative ease of detection [135]. This may contribute to dietary shifts in predators such as reducing their consumption of alternative prey and livestock [135]. Large-bodied omnivores have also been shown to contribute to natural disturbance regimes by increasing natural micro-disturbances in moorland landscapes, potentially facilitating vegetation recovery [136]. They can also facilitate plant long-distance dispersal, potentially playing an important role in enhancing ecosystem connectivity (but also facilitating plant invasions) [137].		

References

1. Angerbjörn A *et al.* 2013 Carnivore conservation in practice: replicated management actions on a large spatial scale. *Journal of Applied Ecology* **50**, 59–67.

2. López-Bao JV, Rodríguez A, Palomares F. 2008 Behavioural response of a trophic specialist, the Iberian lynx, to supplementary food: patterns of food use and implications for conservation. *Biological Conservation* **141**, 1857–1867.

3. Sorensen A, van Beest FM, Brook RK. 2014 Impacts of wildlife baiting and supplemental feeding on infectious disease transmission risk: a synthesis of knowledge. *Preventive veterinary medicine* **113**, 356–363.

4. Robb GN, McDonald RA, Chamberlain DE, Bearhop S. 2008 Food for thought: supplementary feeding as a driver of ecological change in avian populations. *Frontiers in Ecology and the Environment* **6**, 476–484.

5. Redpath SM, Thirgood SJ, Leckie FM. 2001 Does supplementary feeding reduce predation of red grouse by hen harriers? *Journal of Applied Ecology* **38**, 1157–1168.

6. Van Beest FM, Loe LE, Mysterud A, Milner JM. 2010 Comparative space use and habitat selection of moose around feeding stations. *Journal of Wildlife Management* **74**, 219–227.

7. Selva N, Berezowska-Cnota T, Elguero-Claramunt I. 2014 Unforeseen effects of supplementary feeding: ungulate baiting sites as hotspots for ground-nest predation. *PLoS One* **9**, e90740.

8. Selva N, Teitelbaum CS, Sergiel A, Zwijacz-Kozica T, Zięba F, Bojarska K, Mueller T. 2017 Supplementary ungulate feeding affects movement behavior of brown bears. *Basic and Applied Ecology* **24**, 68–76.

9. Jerina K. 2012 Roads and supplemental feeding affect home-range size of Slovenian red deer more than natural factors. *Journal of Mammalogy* **93**, 1139–1148.

10. Jones JD, Kauffman MJ, Monteith KL, Scurlock BM, Albeke SE, Cross PC. 2014 Supplemental feeding alters migration of a temperate ungulate. *Ecological Applications* **24**, 1769–1779.

11. Fischer J, Lindenmayer DB. 2000 An assessment of the published results of animal relocations. *Biological conservation* **96**, 1–11.

12. Johnson WE *et al.* 2010 Genetic restoration of the Florida panther. *Science* **329**, 1641–1645.

13. Schaub M, Zink R, Beissmann H, Sarrazin F, Arlettaz R. 2009 When to end releases in reintroduction programmes: demographic rates and population viability analysis of bearded vultures in the Alps. *Journal of Applied Ecology* **46**, 92–100.

14. Allesina S, Pascual M. 2008 Network structure, predator–prey modules, and stability in large food webs. *Theoretical Ecology* **1**, 55–64.

15. Chesson P, Huntly N. 1997 The roles of harsh and fluctuating conditions in the dynamics of ecological communities. *The American Naturalist* **150**, 519–553.

16. McCann KS. 2000 The diversity–stability debate. *Nature* **405**, 228.

17. Abrams PA, Matsuda H. 1997 Prey adaptation as a cause of predator-prey cycles. *Evolution* **51**, 1742–1750.

18. Seddon PJ. 1999 Persistence without intervention: assessing success in wildlife reintroductions. *Trends in Ecology & Evolution* **14**, 503.

19. Ripple WJ *et al.* 2014 Status and ecological effects of the world's largest carnivores. *Science* **343**, 1241484.

20. Ripple WJ *et al.* 2015 Collapse of the world's largest herbivores. *Science Advances* **1**, e1400103.

21. Liberg O, Chapron G, Wabakken P, Pedersen HC, Hobbs NT, Sand H. 2012 Shoot, shovel and shut up: cryptic poaching slows restoration of a large carnivore in Europe. pp. 910–915. The Royal Society.

22. Kuijper D, Sahlén E, Elmhagen B, Chamaillé-Jammes S, Sand H, Lone K, Cromsigt J. 2016 Paws without claws? Ecological effects of large carnivores in anthropogenic landscapes. p. 20161625. The Royal Society.

23. Côté SD, Rooney TP, Tremblay J-P, Dussault C, Waller DM. 2004 Ecological impacts of deer overabundance. *Annu. Rev. Ecol. Evol. Syst.* **35**, 113–147.

24. Milner JM, Nilsen EB, Andreassen HP. 2007 Demographic side effects of selective hunting in ungulates and carnivores. *Conservation Biology* **21**, 36–47.

25. Van de Walle J, Pigeon G, Zedrosser A, Swenson JE, Pelletier F. 2018 Hunting regulation favors slow life histories in a large carnivore. *Nature communications* **9**, 1100.

26. Ciuti S, Northrup JM, Muhly TB, Simi S, Musiani M, Pitt JA, Boyce MS. 2012 Effects of humans on behaviour of wildlife exceed those of natural predators in a landscape of fear. *PloS one* **7**, e50611.

27. Dorresteijn I, Schultner J, Nimmo DG, Fischer J, Hanspach J, Kuemmerle T, Kehoe L, Ritchie EG. 2015 Incorporating anthropogenic effects into trophic ecology: predator–prey interactions in a human-dominated landscape. p. 20151602. The Royal Society.

28. Kutal M, Váňa M, Suchomel J, Chapron G, López-Bao JV. 2016 Trans-boundary edge effects in the western Carpathians: The influence of hunting on large carnivore occupancy. *PloS one* **11**, e0168292.

29. Lewin W-C, Arlinghaus R, Mehner T. 2006 Documented and potential biological impacts of recreational fishing: insights for management and conservation. *Reviews in Fisheries Science* **14**, 305–367.

30. Roff DA. 1983 An allocation model of growth and reproduction in fish. *Canadian Journal of Fisheries and Aquatic Sciences* **40**, 1395–1404.

31. Wilberg MJ, Bence JR, Eggold BT, Makauskas D, Clapp DF. 2005 Yellow perch dynamics in southwestern Lake Michigan during 1986–2002. *North American Journal of Fisheries Management* **25**, 1130–1152.

32. Pinnegar J *et al.* 2000 Trophic cascades in benthic marine ecosystems: lessons for fisheries and protected-area management. *Environmental Conservation* **27**, 179–200.

33. Cortes-Avizanda A, Blanco G, DeVault TL, Markandya A, Virani MZ, Brandt J, Donazar JA. 2016 Supplementary feeding and endangered avian scavengers: benefits, caveats, and controversies. *FRONTIERS IN ECOLOGY AND THE ENVIRONMENT* **14**, 191–199. (doi:10.1002/fee.1257)

34. Lieury N, Gallardo M, Ponchon C, Besnard A, Millon A. 2015 Relative contribution of local demography and immigration in the recovery of a geographically-isolated population of the endangered Egyptian vulture. *Biological Conservation* **191**, 349–356.

35. Margalida A, Colomer M, Oro D. 2014 Man-induced activities modify demographic parameters in a long-lived species: effects of poisoning and health policies. *Ecological Applications* **24**, 436–444.

36. Cortés-Avizanda A, Jovani R, Carrete M, Donázar J. 2012 Resource unpredictability promotes species diversity and coexistence in an avian scavenger guild: a field experiment. *Ecology* **93**, 2570–2579.

37. Cortés-Avizanda A, Selva N, Carrete M, Donázar JA. 2009 Effects of carrion resources on herbivore spatial distribution are mediated by facultative scavengers. *Basic and Applied Ecology* **10**, 265–272.

38. Monsarrat S, Benhamou S, Sarrazin F, Bessa-Gomes C, Bouten W, Duriez O. 2013 How predictability of feeding patches affects home range and foraging habitat selection in avian social scavengers? *PloS one* **8**, e53077.

39. Selva N, Jedrzejewska B, Jedrzejewski W, Wajrak A. 2003 Scavenging on European bison carcasses in Bialowieza primeval forest (eastern Poland). *Ecoscience* **10**, 303–311.

40. Ray R-R, Seibold H, Heurich M. 2014 Invertebrates outcompete vertebrate facultative scavengers in simulated lynx kills in the Bavarian Forest National Park, Germany. *Animal Biodiversity and Conservation* **37**, 77–88.

41. Lassauce A, Paillet Y, Jactel H, Bouget C. 2011 Deadwood as a surrogate for forest biodiversity: meta-analysis of correlations between deadwood volume and species richness of saproxylic organisms. *Ecological Indicators* **11**, 1027–1039.

42. Seibold S, Bässler C, Brandl R, Gossner MM, Thorn S, Ulyshen MD, Müller J. 2015 Experimental studies of dead-wood biodiversity—a review identifying global gaps in knowledge. *Biological Conservation* **191**, 139–149.

43. Bergman K-O, Jansson N, Claesson K, Palmer MW, Milberg P. 2012 How much and at what scale? Multiscale analyses as decision support for conservation of saproxylic oak beetles. *Forest Ecology and Management* **265**, 133–141.

44. Ranius T, Johansson V, Schroeder M, Caruso A. 2015 Relative importance of habitat characteristics at multiple spatial scales for wood-dependent beetles in boreal forest. *Landscape ecology* **30**, 1931–1942.

45. Dale VH *et al.* 2001 Climate change and forest disturbances: climate change can affect forests by altering the frequency, intensity, duration, and timing of fire, drought, introduced species, insect and pathogen outbreaks, hurricanes, windstorms, ice storms, or landslides. *BioScience* **51**, 723–734.

46. Bowen ME, McAlpine CA, House AP, Smith GC. 2007 Regrowth forests on abandoned agricultural land: a review of their habitat values for recovering forest fauna. *Biological Conservation* **140**, 273–296.

47. Queiroz C, Beilin R, Folke C, Lindborg R. 2014 Farmland abandonment: threat or opportunity for biodiversity conservation? A global review. *Frontiers in Ecology and the Environment* **12**, 288–296.

48. Navarro LM, Pereira HM. 2015 Rewilding abandoned landscapes in Europe. In *Rewilding European Landscapes*, pp. 3–23. Springer.

49. van der Zanden EH, Verburg PH, Schulp CJ, Verkerk PJ. 2017 Trade-offs of European agricultural abandonment. *Land Use Policy* **62**, 290–301.

50. Ceausu S, Hofmann M, Navarro LM, Carver S, Verburg PH, Pereira HM. 2015 Mapping opportunities and challenges for rewilding in Europe. *CONSERVATION BIOLOGY* **29**, 1017–1027. (doi:10.1111/cobi.12533)

51. Canessa S, Oneto F, Ottonello D, Arillo A, Salvidio S. 2013 Land abandonment may reduce disturbance and affect the breeding sites of an endangered amphibian in northern Italy. *Oryx* **47**, 280–287.

52. Moreira F, Rego FC, Ferreira PG. 2001 Temporal (1958–1995) pattern of change in a cultural landscape of northwestern Portugal: implications for fire occurrence. *Landscape Ecology* **16**, 557–567.

53. Albrecht J, Berens DG, Blüthgen N, Jaroszewicz B, Selva N, Farwig N. 2013 Logging and forest edges reduce redundancy in plant–frugivore networks in an old-growth European forest. *Journal of Ecology* **101**, 990–999.

54. Paillet Y *et al.* 2010 Biodiversity differences between managed and unmanaged forests: meta-analysis of species richness in Europe. *Conservation biology* **24**, 101–112.

55. Kusumoto B, Shiono T, Miyoshi M, Maeshiro R, Fujii S, Kuuluvainen T, Kubota Y. 2015 Functional response of plant communities to clearcutting: management impacts differ between forest vegetation zones. *Journal of applied ecology* **52**, 171–180.

56. Fahrig L. 2017 Ecological responses to habitat fragmentation per se. *Annual Review of Ecology, Evolution, and Systematics* **48**, 1–23.

57. Pfeifer M *et al.* 2017 Creation of forest edges has a global impact on forest vertebrates. *Nature* **551**, 187.

58. Schadt S, Knauer F, Kaczensky P, Revilla E, Wiegand T, Trepl L. 2002 Rule-based assessment of suitable habitat and patch connectivity for the Eurasian lynx. *Ecological Applications* **12**, 1469–1483.

59. Schadt S *et al.* 2002 Assessing the suitability of central European landscapes for the reintroduction of Eurasian lynx. *Journal of Applied Ecology* **39**, 189–203.

60. Grashof-Bokdam C. 1997 Forest species in an agricultural landscape in the Netherlands: effects of habitat fragmentation. *Journal of Vegetation Science* **8**, 21–28.

61. Fahrig L. 1997 Relative effects of habitat loss and fragmentation on population extinction. *The Journal of Wildlife Management* , 603–610.

62. Bebi P, Kulakowski D, Rixen C. 2009 Snow avalanche disturbances in forest ecosystems—State of research and implications for management. *Forest ecology and Management* **257**, 1883–1892.

63. Kulakowski D, Rixen C, Bebi P. 2006 Changes in forest structure and in the relative importance of climatic stress as a result of suppression of avalanche disturbances. *Forest Ecology and Management* **223**, 66–74.

64. Rixen C, Haag S, Kulakowski D, Bebi P. 2007 Natural avalanche disturbance shapes plant diversity and species composition in subalpine forest belt. *Journal of Vegetation Science* **18**, 735–742.

65. Garcia-Gonzalez R, Cuartas P. 1996 Trophic utilization of a montane/subalpine forest by chamois (Rupicapra pyrenaica) in the Central Pyrenees. *Forest Ecology and Management* **88**, 15–23.

66. Fuhlendorf SD, Engle DM, Kerby J, Hamilton R. 2009 Pyric herbivory: rewilding landscapes through the recoupling of fire and grazing. *Conservation Biology* **23**, 588–598.

67. Sitters H, Di Stefano J, Christie F, Swan M, York A. 2016 Bird functional diversity decreases with time since disturbance: Does patchy prescribed fire enhance ecosystem function? *Ecological applications* **26**, 115–127.

68. Bowman DM, Perry GL, Higgins SI, Johnson CN, Fuhlendorf SD, Murphy BP. 2016 Pyrodiversity is the coupling of biodiversity and fire regimes in food webs. *Phil. Trans. R. Soc. B* **371**, 20150169.

69. Karavani A, Boer MM, Baudena M, Colinas C, Díaz-Sierra R, Pemán J, Luis M, Enríquez-de-Salamanca Á, Resco de Dios V. 2018 Fire-induced deforestation in drought-prone Mediterranean forests: drivers and unknowns from leaves to communities. *Ecological Monographs*

70. Harper AR, Doerr SH, Santin C, Froyd CA, Sinnadurai P. 2018 Prescribed fire and its impacts on ecosystem services in the UK. *Science of The Total Environment* **624**, 691–703.

71. Alba C, Skálová H, McGregor KF, D'antonio C, Pyšek P. 2015 Native and exotic plant species respond differently to wildfire and prescribed fire as revealed by meta-analysis. *Journal of vegetation science* **26**, 102–113.

72. Thom D, Seidl R. 2016 Natural disturbance impacts on ecosystem services and biodiversity in temperate and boreal forests. *Biological Reviews* **91**, 760–781.

73. Ward J. 1998 Riverine landscapes: biodiversity patterns, disturbance regimes, and aquatic conservation. *Biological conservation* **83**, 269–278.

74. Leigh C, Sheldon F, Kingsford RT, Arthington AH. 2010 Sequential floods drive 'booms' and wetland persistence in dryland rivers: a synthesis. *Marine and Freshwater Research* **61**, 896–908.

75. Turić N, Temunović M, Radović A, Vignjević G, Sudarić Bogojević M, Merdić E. 2015 Flood pulses drive the temporal dynamics of assemblages of aquatic insects (Heteroptera and Coleoptera) in a temperate floodplain. *Freshwater biology* **60**, 2051–2065.

76. Reiley BM, Benson TJ, Everitts J, Bednarz JC. 2017 Does flooding effect the apparent survival and body condition of a ground foraging migrant passerine? *PloS one* **12**, e0175179.

77. Jardine TD, Pusey BJ, Hamilton SK, Pettit NE, Davies PM, Douglas MM, Sinnamon V, Halliday IA, Bunn SE. 2012 Fish mediate high food web connectivity in the lower reaches of a tropical floodplain river. *Oecologia* **168**, 829–838.

78. Ballinger A, Lake P. 2006 Energy and nutrient fluxes from rivers and streams into terrestrial food webs. *Marine and Freshwater Research* **57**, 15–28.

79. Whited DC, Lorang MS, Harner MJ, Hauer FR, Kimball JS, Stanford JA. 2007 Climate, hydrologic disturbance, and succession: drivers of floodplain pattern. *Ecology* **88**, 940–953.

80. Johnson SE, Amatangelo KL, Townsend PA, Waller DM. 2016 Large, connected floodplain forests prone to flooding best sustain plant diversity. *Ecology* **97**, 3019–3030.

81. Thomaz SM, Bini LM, Bozelli RL. 2007 Floods increase similarity among aquatic habitats in river-floodplain systems. *Hydrobiologia* **579**, 1–13.

82. Reich P, Lake P. 2015 Extreme hydrological events and the ecological restoration of flowing waters. *Freshwater biology* **60**, 2639–2652.

83. Bednarek AT. 2001 Undamming rivers: a review of the ecological impacts of dam removal. *Environmental management* **27**, 803–814.

84. Kiernan JD, Moyle PB, Crain PK. 2012 Restoring native fish assemblages to a regulated California stream using the natural flow regime concept. *Ecological Applications* **22**, 1472–1482.

85. Robinson CT. 2012 Long-term changes in community assembly, resistance, and resilience following experimental floods. *Ecological Applications* **22**, 1949–1961.

86. Tompkins DM, Dunn AM, Smith MJ, Telfer S. 2011 Wildlife diseases: from individuals to ecosystems. *Journal of Animal Ecology* **80**, 19–38.

87. Brunet J, Bukina Y, Hedwall P-O, Holmström E, von Oheimb G. 2014 Pathogen induced disturbance and succession in temperate forests: evidence from a 100-year data set in southern Sweden. *Basic and applied ecology* **15**, 114–121.

88. Jones ME *et al.* 2007 Conservation management of Tasmanian devils in the context of an emerging, extinction-threatening disease: devil facial tumor disease. *EcoHealth* **4**, 326–337.

89. Smith KF, Behrens MD, Sax DF. 2009 Local scale effects of disease on biodiversity. *EcoHealth* **6**, 287–295.

90. Ligon FK, Dietrich WE, Trush WJ. 1995 Downstream ecological effects of dams. *BioScience* **45**, 183–192.

91. Power ME, Dietrich WE, Finlay JC. 1996 Dams and downstream aquatic biodiversity: potential food web consequences of hydrologic and geomorphic change. *Environmental management* **20**, 887–895.

92. Kornis MS, Weidel BC, Powers SM, Diebel MW, Cline TJ, Fox JM, Kitchell JF. 2015 Fish community dynamics following dam removal in a fragmented agricultural stream. *Aquatic Sciences* **77**, 465–480.

93. Magilligan F, Graber B, Nislow K, Chipman J, Sneddon C, Fox C. 2016 River restoration by dam removal: Enhancing connectivity at watershed scales. *Elem Sci Anth* **4**.

94. Stanley EH, Doyle MW. 2003 Trading off: the ecological effects of dam removal. *Frontiers in Ecology and the Environment* **1**, 15–22.

95. Lundberg J, Moberg F. 2003 Mobile link organisms and ecosystem functioning: implications for ecosystem resilience and management. *Ecosystems* **6**, 0087–0098.

96. Bauer S, Hoye BJ. 2014 Migratory animals couple biodiversity and ecosystem functioning worldwide. *Science* **344**, 1242552.

97. Torres A, Jaeger JA, Alonso JC. 2016 Assessing large-scale wildlife responses to human infrastructure development. *Proceedings of the National Academy of Sciences* **113**, 8472–8477.

98. Benítez-López A, Alkemade R, Verweij PA. 2010 The impacts of roads and other infrastructure on mammal and bird populations: a meta-analysis. *Biological conservation* **143**, 1307–1316.

99. Hanski I, Ovaskainen O. 2000 The metapopulation capacity of a fragmented landscape. *Nature* **404**, 755.

100. Allan BF, Keesing F, Ostfeld RS. 2003 Effect of forest fragmentation on Lyme disease risk. *Conservation Biology* **17**, 267–272.

101. Tucker MA *et al.* 2018 Moving in the Anthropocene: Global reductions in terrestrial mammalian movements. *Science* **359**, 466–469.

102. Leibold MA *et al.* 2004 The metacommunity concept: a framework for multi-scale community ecology. *Ecology letters* **7**, 601–613.

103. Skuban M, Find'o S, Kajba M, Koreň M, Chamers J, Antal V. 2017 Effects of roads on brown bear movements and mortality in Slovakia. *European Journal of Wildlife Research* **63**, 82.

104. Scoccianti C. 2006 Rehabilitation of habitat connectivity between two important marsh areas divided by a major road with heavy traffic. *Acta Herpetologica* **1**, 77–79.

105. Hassell JM, Begon M, Ward MJ, Fèvre EM. 2017 Urbanization and disease emergence: Dynamics at the wildlife–livestock–human interface. *Trends in ecology & evolution* **32**, 55–67.

106. Fahrig L. 2003 Effects of habitat fragmentation on biodiversity. *Annual review of ecology, evolution, and systematics* **34**, 487–515.

107. Bellard C, Cassey P, Blackburn TM. 2016 Alien species as a driver of recent extinctions. *Biology letters* **12**, 20150623.

108. Ehrenfeld JG. 2010 Ecosystem consequences of biological invasions. *Annual review of ecology, evolution, and systematics* **41**, 59–80.

109. Brooks ML, D'antonio CM, Richardson DM, Grace JB, Keeley JE, DiTomaso JM, Hobbs RJ, Pellant M, Pyke D. 2004 Effects of invasive alien plants on fire regimes. *AIBS Bulletin* **54**, 677–688.

110. Dukes JS, Mooney HA. 2004 Disruption of ecosystem processes in western North America by invasive species. *Revista chilena de historia natural* **77**, 411–437.

111. Gandhi KJ, Herms DA. 2010 Direct and indirect effects of alien insect herbivores on ecological processes and interactions in forests of eastern North America. *Biological Invasions* **12**, 389–405.

112. Herms DA, McCullough DG. 2014 Emerald ash borer invasion of North America: history, biology, ecology, impacts, and management. *Annual review of entomology* **59**, 13–30.

113. Strayer DL, Eviner VT, Jeschke JM, Pace ML. 2006 Understanding the long-term effects of species invasions. *Trends in ecology & evolution* **21**, 645–651.

114. Croll DA, Maron JL, Estes JA, Danner EM, Byrd GV. 2005 Introduced predators transform subarctic islands from grassland to tundra. *Science* **307**, 1959–1961.

115. Manchester SJ, Bullock JM. 2000 The impacts of non-native species on UK biodiversity and the effectiveness of control. *Journal of Applied Ecology* **37**, 845–864.

116. Kettenring KM, Adams CR. 2011 Lessons learned from invasive plant control experiments: a systematic review and meta-analysis. *Journal of applied ecology* **48**, 970–979.

117. Estes JA *et al.* 2011 Trophic downgrading of planet Earth. *science* **333**, 301–306.

118. Allen BL *et al.* 2017 Can we save large carnivores without losing large carnivore science? *Food Webs* **12**, 64–75.

119. Malhi Y, Doughty CE, Galetti M, Smith FA, Svenning J-C, Terborgh JW. 2016 Megafauna and ecosystem function from the Pleistocene to the Anthropocene. *Proceedings of the National Academy of Sciences* **113**, 838–846.

120. Svenning J-C *et al.* 2016 Science for a wilder Anthropocene: Synthesis and future directions for trophic rewilding research. *Proceedings of the National Academy of Sciences* **113**, 898–906.

121. Ripple WJ, Beschta RL. 2012 Trophic cascades in Yellowstone: the first 15 years after wolf reintroduction. *Biological Conservation* **145**, 205–213.

122. Palomares F, Caro TM. 1999 Interspecific killing among mammalian carnivores. *The American Naturalist* **153**, 492–508.

123. Wilmers CC, Crabtree RL, Smith DW, Murphy KM, Getz WM. 2003 Trophic facilitation by introduced top predators: grey wolf subsidies to scavengers in Yellowstone National Park. *Journal of Animal Ecology* **72**, 909–916.

124. McCauley DJ, Young HS, Dunbar RB, Estes JA, Semmens BX, Micheli F. 2012 Assessing the effects of large mobile predators on ecosystem connectivity. *Ecological Applications* **22**, 1711–1717.

125. Hopcraft JGC, Olff H, Sinclair A. 2010 Herbivores, resources and risks: alternating regulation along primary environmental gradients in savannas. *Trends in Ecology & Evolution* **25**, 119–128.

126. Bakker ES, Gill JL, Johnson CN, Vera FW, Sandom CJ, Asner GP, Svenning J-C. 2016 Combining paleo-data and modern exclosure experiments to assess the impact of megafauna extinctions on woody vegetation. *Proceedings of the National Academy of Sciences* **113**, 847–855.

127. Ford AT, Goheen JR, Otieno TO, Bidner L, Isbell LA, Palmer TM, Ward D, Woodroffe R, Pringle RM. 2014 Large carnivores make savanna tree communities less thorny. *Science* **346**, 346–349.

128. Fahrig L, Baudry J, Brotons L, Burel FG, Crist TO, Fuller RJ, Sirami C, Siriwardena GM, Martin J. 2011 Functional landscape heterogeneity and animal biodiversity in agricultural landscapes. *Ecology letters* **14**, 101–112.

129. Pires MM, Guimarães PR, Galetti M, Jordano P. 2018 Pleistocene megafaunal extinctions and the functional loss of long-distance seed-dispersal services. *Ecography* **41**, 153–163.

130. Root-Bernstein M, Svenning J-C. 2017 Restoring connectivity between fragmented woodlands in Chile with a reintroduced mobile link species. *Perspectives in Ecology and Conservation* **15**, 292–299.

131. DeVault TL, Beasley JC, Olson ZH, Moleón M, Carrete M, Margalida A, Sánchez-Zapata JA. 2016 Ecosystem services provided by avian scavengers. *Ecosystem services provided by birds* , 235–270.

132. Moleon M, Sanchez-Zapata JA, Margalida A, Carrete M, Owen-Smith N, Donazar JA. 2014 Humans and scavengers: the evolution of interactions and ecosystem services. *BioScience* **64**, 394–403.

133. Moleón M, Sánchez-Zapata JA, Selva N, Donázar JA, Owen-Smith N. 2014 Inter-specific interactions linking predation and scavenging in terrestrial vertebrate assemblages. *Biological Reviews* **89**, 1042–1054.

134. Pain DJ *et al.* 2003 Causes and effects of temporospatial declines of Gyps vultures in Asia. *Conservation Biology* **17**, 661–671.

135. Mori E, Benatti L, Lovari S, Ferretti F. 2017 What does the wild boar mean to the wolf? *European journal of wildlife research* **63**, 9.

136. Sandom CJ, Hughes J, Macdonald DW. 2013 Rewilding the Scottish Highlands: do wild boar, Sus scrofa, use a suitable foraging strategy to be effective ecosystem engineers? *Restoration Ecology* **21**, 336–343.

137. Dovrat G, Perevolotsky A, Ne’eman G. 2012 Wild boars as seed dispersal agents of exotic plants from agricultural lands to conservation areas. *Journal of Arid Environments* **78**, 49–54.
